# Advanced Genetic and Molecular Techniques for Improving Biocontrol Traits of Entomopathogenic Nematodes

**DOI:** 10.3390/ijms27198596

**Published:** 2026-09-25

**Authors:** Mahfouz M. M. Abd-Elgawad

**Affiliations:** Plant Pathology Department, National Research Centre, El-Behooth St., Dokki, Giza 12622, Egypt; mahfouzian2000@yahoo.com

**Keywords:** biocontrol traits, biotic/abiotic factors, crop pests, insecticidal nematodes, strain improvement

## Abstract

Entomopathogenic nematodes (EPNs) of the genera *Heterorhabditis* and *Steinernema* are insecticidal agents used against diverse groups of key insect pests. Yet, their application cost and reliability frequently relegate them as niche products in the biopesticide market. A full spectrum of developments is being addressed to solve the two issues. Among them, molecular and non-molecular methods play a significant role in improving their biocontrol traits. Relevant programs usually comprise selection, hybridization, and/or mutagenesis/genetic engineering techniques. Progress to boost EPN-biocontrol traits like infectivity, virulence, survival/persistence, and co-optimization of EPNs and their symbionts is discussed herein, showing possible molecular processes as bases for further optimization. Trait improvement may also impact other parts of the cascade that lead to positive outputs such as increasing EPN efficiency or negative trade-offs like decreased baseline fitness. Thus, understanding related molecular processes would help to exploit/strengthen positive ones and fix weaknesses. The purposes of advanced molecular techniques like RNA interference, qPCR, CRISPR-Cas9, and RNA sequencing and their contribution in EPN improvement, as well as their main limitation and validation, are presented. Also, ligand–target interactions of relevant symbionts are amplified to be scalable for efficient exploitation of their secondary metabolites, which are toxic to key hosts. Using reverse transcription quantitative PCR (RT-qPCR) gave molecular cues for understanding how adding dimethyl sulfoxide to the culture medium can boost commercial EPN production, a novel technology for increasing such production. Other molecular cues of certain plant volatiles demonstrated increased EPN efficacy by directing EPN behavior and improving infectivity. The pros and cons of traditional molecular techniques applied on EPNs are presented for their optimal usage. Yet, advanced molecular tools like qPCR analysis combined with traditional ones can offer novel insights to elucidate complicated soil biotic interactions and detect abiotic elements that modify EPN effectiveness. Modern techniques like sequencing of the whole nuclear and mitochondrial genome have rightly resolved *Heterorhabditis* spp. phylogenetic relationships, avoiding former inconsistent tree topologies mostly due to using different gene markers. Other knowledge gaps along with a forward-looking roadmap to catalysts for future studies over the next years are highlighted. Expanding advanced molecular techniques such as metabarcoding can offer perfect EPN uses and secure the best EPN–host matching for economical and sustainable agricultural practices.

## 1. Introduction

Staggering figures of crop losses due to pests [1] coupled with accelerated concern over chemical pesticides [2] have created markets for biopesticide products worldwide. Growers and stakeholders have applied many safe methods to minimize these crop losses, one of which is using insecticidal nematodes, commonly known as entomopathogenic nematodes (EPNs). They possess many attributes of an ideal biocontrol agent (BCA). They are extraordinarily lethal, with most insects being killed within 36 h. Dozens of important insect pests are susceptible, yet non-target populations are undisturbed [3,4]. Despite their high pathogenicity to insects, their lack of threat to the environment led the U.S. Environmental Protection Agency to exempt nematodes from registration requirements; prior to this, many countries provided a similar exemption. This does not negate that genetically modified nematodes or their engineered symbionts can raise case-specific biosafety and regulatory concerns. Generally, their high degree of safety means that unlike chemicals, or even *Bacillus thuringiensis* Berliner (Caryophanales: Bacillaceae), EPN applications do not require masks or other safety equipment, and re-entry time, residues, pollution, and pollinator toxicity are not issues. Nematodes do not require specialized application equipment, as they are compatible with existing equipment, including pressurized, mist, electrostatic, fan, and aerial sprayers. Application via irrigation has further improved grower acceptance [5]. Some researchers believe that evaluating the use of EPNs solely based on their control of insect pests underestimates their value. That is because EPNs can control other pests like plant-parasitic nematodes (PPNs), e.g., serious root-knot nematodes [6,7]. This view is materialized and confirmed when both insect and nematode pests are simultaneously present in a field [8].

Therefore, EPNs of the genera *Heterorhabditis* Poinar (Rhabditida: Heterorhabditidae) and *Steinernema* Travassos (Rhabditida: Steinernematidae) are being extensively investigated and most often applied as BCAs. Meaningful applications comprise the association of these two genera with symbiotic bacterial species of the genera *Photorhabdus* (Enterobacterales: Morganellaceae) and *Xenorhabdus* (Enterobacterales: Morganellaceae), respectively, to facilitate pathogenesis. The nematode in the form of its only free-living stage—called a dauer or infective juvenile (IJ)—search, invade, and enter its insect host. Within the host body, IJs release their mutualistic bacteria via EPN regurgitation and defecation. Usually, the symbionts for species of *Photorhabdus*–*Heterorhabditis* or *Xenorhabdus*–*Steinernema* complexes cause the mortality of insect hosts shortly via septicemia, within 1–3 days [6]. The bacteria first enforce immune suppression to protect themselves. They then rapidly destroy the host from within using targeted toxins (toxemia) while multiplying unchecked until the entire insect cavity is consumed by the bacterial population (septicemia). After feeding on the tissues of the insect body broken down by the bacteria and on the bacteria, IJs of EPN species develop into adults and reproduce a number of times based on the capacity of the insect body. Differences in the life cycle between species of the two genera are illustrated (Figure 1). Yet, *S*. *hermaphroditum* Stock et al. (Rhabditida: Steinernematidae) has a life cycle similar to that of *Heterorhabditis* species. Eventually, EPNs can complete a few generations before consuming the resources in the host cadaver over several weeks, if food is available [9]. Thereafter, having symbionts in their digestive system, many IJs exit the cadaver to search for new arthropod hosts. The dyad, the EPNs, and their mutualistic bacteria are interdependent. While the symbionts rely on the nematodes to infect a new insect host, they generate antibiotics to prevent secondary host infestations and offer a proper diet to feed the EPN within the insect host.

Despite worldwide recognition that EPNs can offer excellent arthropod pest control, in a few cases, there are two documented reasons why their exploitation is often hindered by growers and stakeholders: cost and effectiveness. Hence, upgrading the share of EPNs in the pesticide market should be based on enhancing their market suitability and boosting their reliability. Such issues are being challenged via various aspects such as improving their mass culture, formulation, and field performance [3,4]. Interestingly, when the available EPN strains or species cannot fulfill the desired levels of efficacy against the target insect species, strain enhancement could be achieved via genetic improvement programs. These programs can involve molecular or non-molecular techniques [11].

Two non-molecular methods, i.e., selection and hybridization, have recently been reviewed as successful approaches to improve desirable traits in EPNs [12]. Directed selection proved effective for boosting desired EPN traits like host finding [13,14], EPN efficacy [15], longevity (with marker-assisted breeding) [16], host pathogenicity [17,18], cold/heat tolerance [19,20], nematicide resistance [21], and cost-effective application rates [22]. In such selective breeding programs, gene pools of the used EPN populations must have not only the desired alleles but also their high heritability values. Otherwise, the absence of genetic variation in resistance to ultraviolet (UV) light can result in the failure of such a breeding program to enhance UV tolerance, as shown in *S*. *carpocapsae* Weiser (Rhabditida: Steinernematidae) [13]. Transferring other desired traits from one strain to another has also been advantageous in *S*. *carpocapsae* for control of the pecan weevil, *Curculio caryae* Horn (Coleoptera: Curculionidae), via hybridization and bacterial transfer [23]. Likewise, hybridization is also a robust technique used to combine beneficial traits, with good heritability in a natural population, to generate high-quality strains of heterorhabditid nematodes. The heat-tolerant *H. bacteriophora* Poinar (Rhabditida: Heterorhabditidae) strain ISS was hybridized with *H. bacteriophora* HP88, where heat tolerance was achieved in the hybrid progeny [24], proved by utilizing a mutant marker isolate (Hp-dpy-2) and backcrossing [19]. Moreover, fitness in the obtained progeny was comparable to the parental strain [24]. Also, improving desiccation tolerance and heat in *H*. *bacteriophora* could be attained using methods of hybridization and selection together [25]. Both molecular and non-molecular genetic methods significantly contributed in the related strain improvement [3,4,12]. However, molecular genetics, which usually apply molecular devices to research EPN genes, can allow for “transgenic” EPN improvements that are not possible with classical methods, i.e., selection and hybridization [26]. Simply, it can extend the EPN improvement beyond the limits of the natural gene constitution.

As the tools/techniques for EPN enhancement are still at a relatively early stage, examples of early and current molecular methods used to advance EPN research and boost EPN efficacy are reported herein. This review lists their merits and demerits for careful and astute exploitation. It highlights ongoing techniques that directly target the EPN genome for biocontrol improvement. It also presents other sophisticated EPN molecular techniques for EPN taxonomy and possible editing of genes for strain improvement. These techniques disclose the related progress achieved hitherto, but they also show that more improved EPN strains have yet to be commercialized under natural settings.

## 2. Development of the Molecular Era for Advancing EPN-Related Research

### 2.1. Initiation of the Evolving Molecular Era for EPNs

There are differences and overlaps, no doubt, in the progress and application of nematode molecular biology research, mostly between researchers in developed and developing countries. Such differences are quite apparent, especially when molecular approaches are still costly. Therefore, we should not be surprised to learn that some researchers are still applying molecular genetics related to the pre-omics era or even relatively old molecular tools and techniques. This does not negate the crucial importance of skill and revision in fine-tuning and developing this science worldwide. So, the demerits of such molecular tools and techniques should always be faced, if applicable.

Protein electrophoresis (general total protein profiling) is the oldest widely applied molecular technique in nematology [27]. Separating protein variants based on their mobility in an electrical field has evolved, where a supporting medium such as starch gel, polyacrylamide gel, or cellulose acetate has been employed. Using such electrophoretic techniques could primarily solve the taxonomic and diagnostic bottleneck, as they are more conservative and accurate than morphological identification. They are still used to measure both enzyme and non-enzyme proteins. For example, the hemolymph of an EPN-infected and uninfected fifth larval instar of both *Spodoptera littoralis* Boisduval (Lepidoptera: Noctuidae) and *Agrotis ipsilon* Hufnagel (Lepidoptera: Noctuidae) was separately used to detect protein bands by polyacrylamide gel electrophoresis (PAGE). This study [28] indicated a protein band characteristic of all tested *Heterorhabditis* isolate-infected insects, with a molecular weight of 35 kilodaltons (KDa), defined as responsible for toxicity (toxic protein).

A modified protein-based method that can advance research on EPNs and their symbiotic bacteria is two-dimensional (2D) gel electrophoresis. It has been used to identify proteins secreted by the nematode-symbiotic bacterium *Photorhabdus asymbiotica* Fischer-Le Saux et al. (Enterobacterales: Morganellaceae). The authors [29] found that the *P*. *asymbiotica* adhesion modification protein (Pam) plays a key role in boosting surface attachment in insect blood. Being associated with exopolysaccharides suggests that Pam may exert its impact via the mediation of extracellular polysaccharide (EPS) properties. The conclusion was that Pam binds EPS and shifts bacterial attachment as a host-interacting protein that is relevant to either human or insect infections. Also, serological analysis comprising an Enzyme-Linked Immunosorbent Assay (ELISA) was implied as another protein-based method for advancing EPN application. The infectivity of two EPN species, *H*. *bacteriophora* and *S*. *karii* Waturu et al. (Rhabditida: Steinernematidae), was not negatively affected by *Bacillus thuringiensis* (*Bt*) cotton, as the ELISA was used to assess the presence of *Bt* protein in roots and soil at the end of the growing season of a *Bt* cotton cultivar and non-*Bt* cotton cultivar [30]. This bodes well in applications of EPNs as BCAs in *Bt* cotton agroecosystems, minimizing the use of chemical pesticides. However, many biochemical techniques have employed allozyme pattern analysis exclusively to identify nematode species. Combined with nematode morphology and morphometrics, common enzymes like esterase, malate dehydrogenase, and superoxide dismutase are often used for more accurate identification than the sole use of morphology [31]. So, separating variant enzyme forms generally via differences in net electrical charge, size, and conformation is still a basic way to identify cryptic species and assess population genetics in many studies [32,33]. Thus, a multilocus enzyme electrophoresis (MEE) technique was applied to examine co-evolutionary relationships between *Steinernema* spp. and their mutualistic bacteria, *Xenorhabdus* spp. (Enterobacterales: Morganellaceae) [34]. Nonetheless, a similar electrophoretic template does not necessarily imply that amino acid sequences are identical. A disadvantage of such a result may mask genetic similarity. Therefore, MEE is sometimes documented by morphological or other molecular techniques [33]. On the other hand, cellulose acetate electrophoresis was employed to distinguish isozymes of eight enzymes in IJs of five isolates related to three EPN species: *S*. *feltiae* Filipjev (Rhabditida: Steinernematidae), *S*. *carpocapsae*, and *S*. *riobrave* Cabanillas, Poinar & Raulston. In this study [35], comparing the relative electrophoretic mobility of the isozymes, one of the eight enzymes (arginine kinase) yielded zymograms that were distinctive for each of the isolates, except for two isolates of *S*. *feltiae*, which had identical banding patterns. The same study [35] recorded that four enzymes (fumarate hydratase, phosphoglucoisomerase, phosphoglucomutase, and 6-phosphogluconate dehydrogenase) showed isozyme banding patterns that were characteristic for all isolates, except for two strains of *S*. *feltiae*, which were identical. Therefore, the authors concluded that cellulose acetate electrophoresis could be advantageous for steinernematids taxonomic identification when a combination of enzymes is utilized. Moreover, cellulose acetate medium has quicker running times and high sensitivity with superior band resolution compared to polyacrylamide or starch. Campos-Herrera and Lacey [36] reported that studies comprising conspecifics or paternity approaches that require the ability to detect individual differences are not suitable for this approach.

### 2.2. The Pre-Omics and Omics Era Using Nucleic Acids

Generally, “omics” is a recent suffix-based term (e.g., genomics [all genes], proteomics [all proteins], and metabolomics [all chemical metabolites of small molecules]) referring to the complete set of objects or items in biology. From such suffix-based terms, the pre-omics era suggests that the set of biological entities in a subject being studied was not holistically and/or simultaneously researched. Rather, the set was addressed via a simplistic approach. Portions of the EPN genome that vary between taxa or even within a taxon, as well as those conserved within a taxon, are practical for identification. In order to carry out analyses on DNA and RNA, it is essential to first recover these molecules from EPN cells/tissues and perform related techniques for the analysis process. As informatory tools, polymerase chain reaction (PCR)-based molecular techniques permit the analysis of DNA markers and genes of individual EPNs. Advantages of such molecular markers are that any of them represent a DNA sequence that is widely diffused throughout the EPN genome. Moreover, the marker demonstrates polymorphism that can be uncovered utilizing molecular techniques. Such markers must also be stable in order to enable their characterization in each EPN developmental stage [10].

The intended regions utilized for EPN characterization and identification are nuclear, ribosomal, and mitochondrial deoxyribonucleic acid (DNA). The cistron of ribosomal DNA (functions as a unit of genetic action defined by the cis–trans complementation test; a structural unit to encode ribosomal RNA [rRNA]) comprises three highly conserved genes (i.e., remain identical/very similar across different EPN species for long periods of time). These are 18S rRNA, 5.8S, and 28S. It contains also highly variable regions such as the internal transcribed spacer (ITS; ITS1 and ITS2), IGS (intergenic spacer), and ETS (external transcribed spacer) regions. Such regions can offer high-resolution species differentiation, evolutionary studies, easy amplification via conserved flanking genes, and high copy numbers for sensitive detection. Consequently, the pattern of resolution of the EPN DNA portions may be utilized via various techniques [31]. These may comprise Restriction Fragment Length Polymorphism (RFLP) [37,38], random-amplified polymorphic DNA (RAPD), Amplified Fragment Length Polymorphism (AFLP), and the use of species-specific primers, which relies on the presence/absence of a PCR amplification product [12,39]. All these methods include PCR followed by electrophoresis, except for RFLP, as PCR may not be required [31]. Surely, using PCR has several merits: mainly, EPNs do not require EPN culturing prior to processing by PCR. Additionally, PCR-based approaches are quick, versatile, and have high sensitivity (accuracy) in solving biological bottlenecks. For example, ITS sequencing has solved unreliable/impossible species identification at the IJ stage. That is because EPNs collected from the field or White traps usually lack the clear adult structures (e.g., spicules in males or vulval flaps in females) required for classical taxonomy. So, sequencing the highly variable but universally targetable ITS region of ribosomal DNA acts as a definitive DNA barcode. It permits unambiguous species-level identification and phylogenetic placement of any EPN life stage. Likewise, the other techniques could effectively resolve biological bottlenecks. In this respect, RFLP utilizes restriction enzymes to cut DNA into distinct patterns, providing stable, reproducible genetic markers to confirm species boundaries and map chromosomal variations without relying on visible traits. So, it avoids the inability to differentiate morphologically identical or cryptic EPN species and strains. Studies are still ongoing using PCR-RFLP, relying on generating many copies of a specific target DNA region by PCR followed by digestion of the amplified ribosomal or mitochondrial PCR products with restriction enzymes to create distinct fragment profiles for prompt EPN identification, of course, without full genome sequencing [37]. In other words, PCR-RFLP involves amplifying specific ribosomal DNA regions (such as internal transcribed spacers [ITS1 and ITS2]) and cutting them with restriction enzymes to create distinct diagnostic fingerprints for strain improvement and identification. RAPD is another extension to the variety of current approaches that exploit polymorphic DNA markers for similar studies on EPNs. It solved the lack of prior genome sequence information for local and novel EPN isolates. Furthermore, RAPD requires no sequence data. It offers quick genetic screening, is low cost, and needs no prior sequence data (applies to anonymous genomes with even limited quantities of DNA) [40]. Yet, it suffers from poor laboratory reproducibility. So, AFLP combines restriction digestion with selective PCR amplification. It allows us to scan hundreds of loci across the EPN genome simultaneously, solving the need for high-resolution genomic profiling to track strain migration, hybridizations, or laboratory selection traits. Strictly speaking, AFLP solved poor reproducibility when analyzing complex genome-wide variations and population structures. On the other hand, using quantitative PCR (qPCR) allows for quick, highly sensitive detection and exact quantification of specific EPN species directly from environmental soil samples or mixed infections inside a host pest. Thus, qPCR negates the need for using direct microscopic observation, which requires considerable expertise to identify species and suffers from a deficiency of definitive morphological features. It also solved another biological bottleneck materialized in avoiding the use of soil baiting with sentinel insects to recover EPNs with its inherited baiting errors. The latter fails to detect EPNs due to incompatible host status of the sentinel, competition between EPNs and other organisms, and phased infectivity exhibited by many EPN populations. The main limitation of qPCR is that it identifies only the species matching the primer or probes that are utilized. Metagenomic methods solve this bottleneck, as they can identify EPN species independently. Nonetheless, taxonomic identification still requires appropriate phylogenetic or reference-based validation.

Contrary to RAPD, robust and reproducible markers like simple sequence repeats (SSRs) and single nucleotide polymorphisms (SNPs) may often replace it. Microsatellites (or SSRs) are short, repeating DNA motifs employed as genetic markers. They were applied to showcase their utility as markers to understand the global diffusion of PPNs and EPNs [41]. Their key merits are high polymorphism and co-dominant inheritance, but their major demerits involve high initial development costs and potential amplification stutter. For example, SSRs need prior genomic sequence data or labor-intensive cloning to design flanking primers for new EPN species; both are time-consuming. On the other hand, SNPs can provide high EPN genomic density and precise resolution. It combined beneficial traits in a single breeding line. Admittedly, both SSRs and SNPs have solved major biological, ecological, and industrial bottlenecks. Marker-assisted selection (MAS) using SNPs enabled breeders to map quantitative trait loci (QTLs) for traits like EPN dual juvenile (DJ) recovery and reproductive potential. This permits targeted molecular-assisted breeding to sustain a high yield of in vitro EPN mass production with stable commercial batches, without losing field efficacy [42]. Thus, Ogaya et al. [42] applied marker-assisted breeding via SNPs associated with reproductive potential, longevity, virulence, and cold tolerance for a collection of *H. bacteriophora* WT inbred lines. Another superior function of SNPs was to use genotyping by sequencing (GBS) to identify specific SNPs linked to critical environmental traits like virulence at extreme temperatures. This technique could rapidly screen and breed climate-resilient strains of *H*. *bacteriophora* for extended survival [16]. Yet, such related techniques suffer from high initial discovery costs and limited reference genomes. Studying EPNs with SNPs involves distinct trade-offs in genetic mapping and strain improvement. On the other hand, AFLP analysis was used to track EPN field populations of *S*. *feltiae* and *H*. *downesi* Stock, Griffin, & Burnell (Rhabditida: Heterorhabditidae) over several years [43]. The authors conducted mesocosm studies of their genotypic persistence and competition against introduced (exotic) *S*. *carpocapsae* and *H*. *megidis* Poinar et al. (Rhabditida: Heterorhabditidae) strains to test predictions for their relative setting, persistence, and introgression in Irish forestry. These EPN species and strains were inundatively applied to pine stumps to control populations of pine weevil, *Hylobius abietis* L. (Coleoptera: Curculionidae), on three sites, where EPN persistence and diffusion were monitored for up to five years. Their findings [43] from the field study affirmed predictions based on previous knowledge of the EPN species and proved the significance of medium-term investigations because a 3-year study would have overestimated the risk of setting exotic species. Eventually, all tested species persisted over a number of seasons, but only *S*. *feltiae* was established. In another study, AFLP analysis was used to detect intra-species diversity. Since *Steinernema monticolum* Stock et al. (Rhabditida: Steinernematidae) is one of the most commonly isolated EPNs from Korea, the diversity of this species was examined. Therefore, single-enzyme AFLP (SE-AFLP) analyses were utilized to distinguish between 32 *S*. *monticolum* populations [44]. Although AFLP yields dominant markers with strict laboratory precision, it does not require prior sequence knowledge. Eventually, it should be quite clear that the aforementioned methods, like RFLP, RAPD, ITS sequencing, qPCR, and metabarcoding, are valuable for identification, population monitoring, and ecological deployment, but they do not directly improve EPN traits. Contrarily, selection, hybridization, RNAi, mutagenesis, transgenesis, and genome editing are used to directly improve EPN traits. Also, the above-mentioned discussion of genetic tools like starch gels, PAGE, RFLP, RAPD, and AFLP might be disproportionate to the “advanced genetic and molecular techniques” addressed herein. Yet, they still represent a cornerstone of molecular advancements via shifting from sole morphology-based identification of EPNs, especially in many developing countries. Pairing traditional EPN morphology with such genetic tools is still ongoing in these countries. This pairing can avoid the misidentification of high intraspecific morphological variability and subjective dependence on specialized expertise. Moreover, even the most recently published results may incorporate qualitative enzymatic screening of morphological traits and 16S rRNA sequencing to characterize EPNs and/or their symbionts [45].

Admittedly, functional validation in EPNs is an imperative component for improving biocontrol traits. It primarily relies on reverse-genetics tools and phenotypic assays to confirm the biological roles of specific genes. Although their main methods may rely on techniques such as RNAi and CRISPR-Cas9 for genome editing, downstream screening via in vivo experimentation and in vitro bioassays are imperative. The latter are used to measure the functional outcomes of genetic alterations, e.g., changes in IJ locomotion and/or development as well as host-finding capabilities and overall virulence against target insect larvae [12,26]. On the other hand, molecular markers are critical tools across EPN breeding, taxonomy, and diagnostics [46]. Basically, they are specific DNA sequences used to identify genetic variations, track inheritance traits, and distinguish between different EPN species/strains. For EPN breeding, marker-assisted selection (MAS) links specific DNA markers to desirable phenotypic traits; e.g., high virulence, heat tolerance, or host-seeking capability [47]. So, they are used to screen and select parental lines, negating the need to wait for lengthy phenotypic trials. Also, these markers help map quantitative trait loci (QTLs) responsible for complex traits and assess the genetic variation within wild or laboratory populations to block inbreeding depression [8]. For EPN taxonomy, molecular markers can define evolutionary relationships, mapping out exactly how different EPN families and species are diverged, avoiding mis-taxonomy based solely on morphology [46]. Moreover, ITS regions, as well as the 18S and 28S rDNA subunits, contain highly conserved sequences punctuated by variable regions. So, they serve as universal barcodes to define species boundaries. Cytochrome c oxidase subunit I, encoded by a specific gene located within the mitochondrial genome, may also be used to differentiate closely related species or distinct geographic populations. As for MAS usage for diagnostic methods, the aforementioned ones like PCR-RFLP and qPCR have already indicated detection and quantification of a specific EPN species directly from complex environmental or soil samples. Occasionally, a species-specific PCR technique is also used via customized primers designed to bind only to the DNA of a single target EPN species. If a PCR band appears, the presence of that exact EPN species is confirmed.

Meanwhile, modern long-read genomics, pangenomics, high-throughput phenotyping, and integrated host–symbiont genomics are the real sophisticated genetic tools. So, they can contribute to solving further outstanding biological bottlenecks for EPN improvement. Opposite to short-read genomics that break DNA into tiny pieces, making it nearly impossible to correctly assemble these repeating loops, long reads span thousands of base pairs continuously. Hence, they generate gapless, telomere-to-telomere reference assemblies [48,49,50]. So, long-read sequencing gives rise to maps of complete gene families with biocontrol-related traits like pathogenicity and stress tolerance. It enables doing more accurate downstream assays based on more complete gene and transposable element predictions [51]. Moreover, long-read metagenomics can sequence an EPN and its internal microbiome simultaneously without misassembling or mixing up shared genetic material. It offers insights into examining the molecular “crosstalk” that allows the nematode and bacteria to cooperate. In this respect, integrated host–symbiont genomics of EPNs and their mutualistic bacterium are necessary to decode such dual parasitism and immunosuppression of insect hosts, enabling the genomic mapping of the specific genes behind the relevant coordinated effectors [52]. While this mapping helps in understanding the symbiotic mutualism, the integrated data form solid bases for right breeding or selecting superior EPN strains for pest management. Moreover, Schwarz et al. [53] assembled genomes of both *S*. *hermaphroditum* and its associated bacteria. Interestingly, they found sequence data for not only its known symbiont, *X*. *griffinae*, but also for eight other bacterial genera. All eight genera have previously been noted to be associated with *Steinernema* species or the EPN *Heterorhabditis* and may constitute a “second bacterial circle” of EPNs.

It should be emphasized that traditional research on genomics often examines complex DNA, where related variations are easily missed. Such defects are due to relying on a single reference genome from just one EPN or a few individuals. Moreover, contrary to high-throughput phenotyping (HTP), traditional manual screening of EPN traits is commonly slow, subjective, and labor-intensive [54]. Factually, HTP—using computer vision, automated microfluidics, and machine learning—is vital for accelerating genetic selection and breeding [54,55]. It can solve biological difficulties via linking genomics to phenomics and optimizing EPN commercial mass production [16,42,47]. In this context, pangenomics can also examine the complete genetic diversity of a population [56]. Hence, a pangenome represents a diverse group of individuals within the same species. Therefore, it supplies a multi-dimensional map to prevent missed unique variants and consequently minimize reference bias. Admittedly, additional biological bottlenecks are yet to be examined and solved via advanced genetic and molecular techniques. Currently, in addition to all such molecular tools, non-molecular ones, field sampling strategies, and laboratory extraction protocols [mine sampling] are used as ecological monitoring devices to detect, quantify, track, and improve biocontrol traits of EPN populations. Surely, each of these approaches does not contribute to strain improvement of EPNs in the same way. Yet, they can be easily arranged to do so in a logical sequence.

### 2.3. Examples of How to Improve Biocontrol Traits of EPNs

In order to evaluate EPN fitness and quality against a targeted insect host, their biocontrol traits should be examined for optimization. Thus, nematologists and molecular biologists try to boost traits like nematode viability (percentage of emerged living infective juveniles), virulence (power to kill), infectivity (power to invade), and reproductive capacity (yield per insect) [57] to upgrade the biocontrol potential of the targeted host. A brief overview of a few key examples of such traits and the existing possibility to boost them is presented herein. They clearly correspond to other traits, as detailed in related references [3,4,12,13].

***Infectivity***: This biocontrol trait is considerably impacted at every step of the infection process. It is initiated by performing host-seeking behaviors so that EPN IJs locate a proper insect host to enter their body. The foraging process of IJs lies between two specific ends of the ambusher–cruiser continuum. The foraging strategies of a few EPN species/strains have been determined, but the relevant practices are mostly modified by other factors like volatile cues released from the host insects and/or plant roots associated with the existing environment, as well as soil qualities like texture, moisture, and temperature [58]. Hence, for boosting EPN infectivity, specific plant volatiles may be used to direct EPN behavior and raise its efficacy [59]. Moreover, studies of the volatilome, which involves the complete set of volatile and semi-volatile compounds generated in the environments that contain EPNs, should be expanded to the entire related ecosystem to draw out correct and reliable conclusions.

It should also be noted that species within *Steinernema* and *Heterorhabditis* differ significantly in their physical entry methods, soil foraging behavior, and cadaver characteristics when infecting a definite insect host. For example, infectivity of *Heterorhabditis* species is usually facilitated by a specialized cuticular dorsal tooth in the IJs (not found in *Steinernema* spp.) that aids in penetrating hard insect cuticles (if penetration did not occur via natural insect openings), along with highly virulent *Photorhabdus* symbiotic bacteria that promptly defeat insect defenses [60].

On the other hand, EPN infectivity of both genera relies heavily on local adaptation, soil moisture and temperature, and target matching (for best EPN species or strain–host matching) rather than a broad rule favoring one entire genus over other. A positive correlation is usually found between the host exposure time and IJ infection rate, but marked infectivity divergences may occur among different EPN species/strains infecting the same host species. Very recently [61], percentages of infection of the greater wax moth *Galleria mellonella* L. (Lepidoptera: Pyralidae) larvae by EPNs found in the soils of carob forests in Tumbes, Peru, were recorded. These were 10, 5, and 30% by *H. bacteriophora* strain 02 and *H. indica* Poinar et al. (Rhabditida: Heterorhabditidae) strains 05 and 06, respectively, after 12 h of exposure. Both morphological and molecular characterizations of these EPNs helped to mark such infectivity divergences. In contrast, multiple cycles of a *Galleria* baiting technique for isolating EPNs from soil samples proved to be promising [62]. The more these EPN extraction cycles run, the better they reveal new samples positive for *Heterorhabditis indica*, a strict indication of more EPN infection [62].

After locating a suitable host, IJs must obtain entry into the hemolymph, release their mutualistic bacteria, overcome the host immune response, kill the host, liberate host tissues and nutrients into a useable form, develop, and reproduce, and eventually, when cadaver resources become limited, the rising generation of nematodes must acquire or associate with their symbiont and emerge as IJs to repeat the cycle [26]. These authors confirmed that any part of these processes and other unrecorded/unknown but related parts could potentially be targeted for genetic improvement and could result in a higher level of infectivity, thus enhancing the efficacy of EPN biocontrol. Key studies have been conducted and reviewed [12,26] to investigate traditional selective improvement of host-seeking behaviors and infectivity, with varying levels of success. For instance, a 72-fold gain in the host-finding ability of a definite *S*. *carpocapsae* strain against the Japanese beetle *Popillia japonica* Newman (Coleoptera: Scarabaeidae) was achieved in laboratory breeding, but no difference in infectivity was detected relative to the wild-type strain under field settings [63]. This is probably due to an efficacy gap between laboratory and field setting, which should be considered. The method imposed an overall fitness cost on the chosen strain. To avoid/lower such defects, a selected strain may be secured via cryopreservation in liquid N_2_ [64] and the strengthening of selection pressure at even intervals [65]. On the contrary, after six generations of selection, a selected strain of *H. bacteriophora* responded considerably better and moved twice as rapidly towards a (*E*)-β-caryophyllene source (emitted by roots of corn plants) than the original strain [66]. In subsequent field tests, this selected strain was significantly more effective than the original strain in suppressing Western corn rootworm populations in plots with a corn variety that releases (*E*)-β-caryophyllene but not in plots with a corn variety that does not emit this root signal. These results demonstrated the great potential of manipulating *H. bacteriophora* to enhance foraging and infectivity with consequently superior biological pest control.

On the other hand, Lu et al. [67] emphasized that the current access to genomic sequence data and putative proteomes offered a large number of genes that could be useful in enhancing EPN infectivity. Examples of such potential targets for enhancement are proteases, protease inhibitors, fatty acid- and retinol-binding proteins (FAR proteins), and the G protein-coupled receptor (GPCR) family. Yet, many other genes in EPN genomes remain to be functionally tested. Various related approaches are being addressed to advance EPN infectivity to elevate EPN efficacy. Some of them take indirect routes. Morris et al. [68] used RNA interference (RNAi), commonly used to study gene function or silence targeted genes, for gene *flp-21* knockdown in *S*. *carpocapsae* IJs. It led to the inhibition of nematode dispersal, host-finding (chemosensory), hyperactive nictation, and jumping behaviors. In other words, gene knockdown did not directly “enhance” the intrinsic infectivity of *S*. *carpocapsae* IJs but indirectly grasped related processes like host-seeking behavior. On the other hand, mutagenesis may be acceptable where a few regulatory genes are in charge of the demanded trait, e.g., infectivity. Surely, molecular studies can fortify mutagenesis by using precise tools such as PCR and synthetic primers to engineer targeted DNA alterations. Thus, relevant applications enable our investigation of protein function, can map genetic pathways, and allow us to develop novel biochemical attributes via mutagenesis. The latter was achieved in mutagenic strains with elevated desiccation tolerance. As boosting EPN desiccation tolerance implies better infectivity in relatively dry conditions, mutants of *H. megidis* (Strain UK211) were isolated with such increased tolerance [69].

Importantly, molecular studies documented that cues liberated by IJs can negatively affect the invasion of root insect herbivores [70]. The authors speculated that one of the compounds released by EPN cadavers (that is, indole) is also generated by some plant species after herbivore damage and has been characterized as a key defense priming signal in the plant [70,71]. Thus, their observation of priming potato plant defenses in response to EPN cues [71] suggested that plants detected this compound as a cue linked to herbivore damage. This has previously been documented as a plant resistance increase following exposure to PPN ascarosides, a type of molecule used in EPN chemical communication as well [71,72]. Hence, plants might recognize broad taxonomically conserved cues that EPNs share with their plant-parasitic relatives, and the observed plant response is an artifact of an over-generalized response to nematodes. Intriguingly, Manosalva et al. [73] stressed that since plants recognize ascarosides as a conserved molecular signature of nematodes, their usage to activate plant immune responses has the potential to boost the economic and ecological sustainability of agriculture. Further genetic and molecular studies are desperately needed to exploit such cues for improving EPN infectivity and/or priming plant defense against pests. This is especially important, as promising results have suggested the importance of volatile compounds in other aspects for biocontrol of insect pests. For instance, earnest attempts to broadly advance biocontrol of *Oides leucomelaena* Weise (Coleoptera: Galerucinae) have molecularly characterized Odorant-Binding Protein (OBP) genes linked to relevant host-seeking behavior [74]. Three antenna-specific OBPs from *O*. *leucomelaena* were obtained, and their high-affinity volatiles were determined. Thus, they offer a theoretical basis for the development of novel biocontrol strategy in subsequent stages. Yet, such a strategy requires further exploring the molecular mechanisms of the chemosensory system in this pest. In parallel, molecular biologists should target chemosensory genes to improve how EPNs detect carbon dioxide, host feces, or cuticular hydrocarbons with consequent elevated infectivity.

***Virulence*:** Research on enhancing EPN virulence has been focusing on selective breeding and isolate/strain screening [12], optimizing symbiotic bacteria, managing evolutionary trade-offs to improve biological pest control efficacy [75], and applying chemical priming like pheromones [76]. Screening of thirty-eight populations of Egyptian *Heterorhabditis indica* for virulence against the most damaging third-instar larvae of the scarab beetle, *Temnorhynchus baal* (Reiche & Saulcy) (Coleoptera: Scarabaeidae), in two soil types from strawberry fields, where this insect persists as a serious pest, was promising. In this study [57], the corrected mortality caused by *H*. *indica* populations in the beetle larvae averaged 99.52 and 98.57% for 15 populations in the sandy soil and 23 in loamy sand soil, respectively. Following the same line of thinking, Campos-Herrera et al. [77] found the *S. feltiae* Koppert population to be the most consistent among the *S. feltiae* populations tested against *Frankliniella occidentalis* Pergande (Thysanoptera: Thripidae) and *Tuta absoluta* Meyrick (Lepidoptera: Gelechiidae). Although *S. feltiae* virulence is superior to other EPN species for controlling the two pests, their study emphasized the significance of intraspecific variability in this virulence against the two pests.

Generally, further or multi-generation selection through hosts or even liquid culture may result in trade-offs such as decreased baseline fitness or unstable trait retention, especially without continuous selection pressure [12]. Such fitness trade-offs were shown by changed virulence, reproduction, dispersal, and storage stability in selective breeding of *S*. *carpocapsae* strains [14,78]. Therefore, as aforementioned with the “infectivity” trait, cryopreservation in liquid N_2_ [64] and strengthening of selection pressure at even intervals [65] may be applied to avoid such negative trade-offs. Baiocchi et al. [26] regarded the issue of maintaining desired characteristics as the biggest challenge for species of EPNs that reproduce sexually. Yet, they speculated that the issue may be overcome through the utilization of advanced methods for genetic improvement as well, e.g., transgenics. Additionally, some studies have explored new avenues to enhance EPN virulence using chemicals. Perier et al. [76] noted that *S. feltiae* have only low to moderate virulence to *C. caryae* larvae. So, they applied pheromone extracts including ascarosides to boost the efficacy of this nematode when used as a BCA against *C. caryae* field populations. Consequently, the effectiveness of *S. feltiae* was considerably enhanced, as measured with reduced *C. caryae* survival when exposed to the pheromone blend, despite unfavorable environmental conditions. Definitely, exposure to ascaroside pheromone blends acted as a behavioral booster for *S. feltiae,* as the molecular signaling role of these pheromones stimulated chemosensory pathways. This stimulation, in turn, significantly enhanced *S. feltiae* mobility, soil dispersal, and host-seeking infectivity, leading to higher mortality rates in the pecan weevil, i.e., elevated virulence [76]. The authors presumed that with such an increased efficacy, utilizing EPNs will reduce reliance on conventional management tactics that use unhealthy chemicals in pecan production, translating into sustainable and eco-friendly agricultural exercises.

These attempts do not negate additional possible contributions of molecular biology in increasing EPN virulence via their mutualistic bacteria. The role of the latter is especially important, as monoxenic IJs carrying their particular bacterial dyad prove drastically higher virulence than axenic (bacteria-free) EPNs. Thus, Kaur et al. [60] documented the major role of these symbionts in upgrading insect toxicity. Within this context, Glazer et al. [75] recently reviewed how EPNs generate and secrete effector molecules during the initial stages of infection to interact with the mechanisms that control the host innate immune action. These EPN effectors can also ease the following release and diffusion of their mutualistic bacteria within the insect host. Therefore, a comprehensive understanding of the underlying virulence mechanisms allowing protection against ecological conditions and insect host immune responses is necessary to realistically improve EPN performance under field settings. This requires an interpretive knowledge of the successive stages in the nematode’s invasion and induction of mortality for the insect host. In *H*. *bacteriophora*, such key stages lie in the IJs’ recovery from developmental arrest and resuming growth and development within the host. Moshayov et al. [79] considered this “recovery” as the first outcome (process) of the host–parasite interaction. As arrested IJs turn into active parasites, this EPN “recovery” largely contributes to their commercial reliability and efficiency in agricultural pest control. Thus, genes involved in the “recovery” process were identified via a large-scale bioassay for recovery, where subtraction libraries of recovering IJs from arrested IJ transcripts were constructed at different time points. The authors [79] found that the majority of the genes characterized as differentially expressed between recovering and developmentally arrested IJs belonged to metabolic pathways. Increased expression levels of 23 selected genes during recovery were proven by quantitative PCR (qPCR). For eight of these genes, transcription silencing in *H*. *bacteriophora* led to a significant decrease in IJ recovery rates, suggesting that these genes are critical to the recovery process. Two of the genes were linked to the insulin-like growth factor-1 (insulin/IGF-1) pathway, renowned to regulate dauer formation in the free-living nematode *Caenorhabditis elegans* Maupas (Rhabditida: Rhabditidae). However, the other six genes were connected to pathways not previously associated with recovery in nematodes. The study [79] suggested that although little is known about parasitism-unique genes, the pathways regulating recovery in *H*. *bacteriophora* involve those operated in *C*. *elegans* and those that might be unique to parasitic nematodes. The latter may be switched on to answer the host signals and qualify the parasite to recognize its insect host. Further studies are needed to explore how such comparative genomics (e.g., *H*. *bacteriophora* versus *C*. *elegans*) and genes with different functions can reshape the relevant biocontrol field. Such investigations should upgrade our knowledge from descriptive metabolite cataloguing toward comprehensive analyses of these successive stages in the nematode’s invasion and induction of insect mortality. They should address future directions for integrating bioactive compound identification of EPNs and their symbionts with pathway elucidation for effective biocontrol application. To this end, Glazer et al. [75] concluded that characterizing the key players that regulate EPN survival, virulence, and immunity could unfailingly result in the evolution of more robust and informative solutions for effective EPN application strategies. They would involve molecular and genetic tools and formulation technologies for reliable EPN usage against pests.

***Survival and Persistence***: Logically, EPNs in their normal soil habitat face many factors—involving chemical, biological, and physical components—that impact their survival and persistence as BCAs. Among these factors, two key components, i.e., desiccation and temperature extremes, can greatly affect EPN activities. Interestingly, EPNs can survive only 2–3 weeks in dry soil, but increasing their ability to survive the environmental factors enhanced their efficacy as BCAs [12]. Because EPNs are limnoterrestrial, they are very sensitive to desiccation. In contrast, commercial production of EPNs relies on desiccation tolerance as a means of prolonging their life for storage and shipment as well. Hence, desiccation tolerance is essential in prolonging the life of EPNs, not only to expand shelf life for the purpose of distribution but also to secure their persistence in the field. Although low water content in the EPN body induces quiescence and prevents bacterial and fungal growth, desiccation is a tough factor that leads to a high EPN mortality rate generally [75].

Various research approaches to enhance EPN survival and persistence have been employed. Mostly, they have addressed stress tolerance (heat and desiccation in addition to UV for above-ground pest control), advanced formulations (polymers, gels, nanoparticles, and emulsions), and genetic strain improvement. Key studies and thematic reviews report these approaches [3,4,12,26,33]. Enhancing desiccation and heat tolerance has mostly relied on selective breeding and/or hybridization as a means for boosting EPN survival [26]. Surely, molecular techniques are being used in EPN breeding and hybridization to track genetic inheritance, confirm successful crosses, and screen for the advantageous biocontrol traits. Such techniques are superior to the ones that rely solely on slow phenotypic assays. Therefore, Perry et al. [80] demonstrated the importance of using molecular knowledge involving genome sequence data and expressed sequence tags to define the underlying genetic elements that influence EPN longevity and stress tolerance. In this respect, EPN groups with innate dehydration strategies survive water loss by entering anhydrobiosis. This process relies on transcriptional activation, the accumulation of protective osmolytes like trehalose and glycerol, and the deployment of structural proteins that shield cellular machinery from collapsing from water loss. To control drying, this group enabled biochemical changes to promote long-term survival. Initially, the related nematodes control the rate of drying before their successful entry into long-term anhydrobiosis, which relies on subsequent biochemical and molecular adaptations [80]. Thus, molecular tracking of phenotypic traits like virulence, stress tolerance, and environmental persistence back to core genetic, biochemical, and physiological pathways to better understand the bases for superior biocontrol traits is necessary. So, comprehensive understanding of desired EPN efficacy is not limited to the above-mentioned EPN effector molecules and symbiotic partnerships only. It also involves stress and virulence interactions. Biochemical changes in IJs that facilitate temperature tolerance include trehalose build-up with heat shock protein (HSP) accumulation (specifically hsp70), playing a crucial role in protein homeostasis and chaperone activity to clear misfolded proteins to enhance their survival under heat stress [81,82]. Hence, pathways involving trehalose and heat shock proteins should be evaluated in future EPN improvement tests to ensure that the virulence traits remain stable under environmental extremes. Moreover, further attention should be given to crosstalk between stress and virulence in EPN settings. For instance, naturally occurring heterorhabditid longevity was firmly and positively correlated to heat tolerance (can withstand temperature of 40 °C for 2 h) and UV [83]. Then, exposure of the nematodes to mild temperature shifts was advantageous during application for EPN reproductive capacity (i.e., recycling potential). Remarkably, Lee et al. [84] characterized shifts in olfactory preferences as a complex interactive role of temperature and IJ age for host-seeking behaviors. In this respect, IJs propagated at lower temperatures more actively seek hosts compared to IJs propagated at higher temperatures. Thus, the related implications could be crucial because olfactory cues are essential for IJs’ successful parasitism, particularly for EPN inoculative application strategies. Yet, Shapiro-Ilan et al. [82] speculated that this paradigm needs to be validated in the field from an applied perspective.

Intriguingly, research on proteome shifts in storage at 9 °C (for up to 9 weeks) and 20 °C (for up to 6 weeks) showed varied responses between *Steinernema* spp. and *Heterorhabditis* spp. Steinernematidae increased their stress response genes and chaperone activities, but Heterorhabditidae elevated their cytoskeleton, cell signaling, and carbon metabolism. The proteins that decreased in abundance to the greatest extent were heat shock and ribosomal proteins and those associated with metabolism. Due to such changes, this study [85] demonstrated a fundamental genetic difference in adapting to increased temperature stress. Overall, EPNs exposed to desiccation experienced various degrees of ultrastructural and molecular shifts comprising trehalose accumulation, expression of Late Embryogenesis Abundant (LEA) protein, heat shock protein, detoxifying mechanisms, and anhydrobiosis [82]. Accordingly, different EPN species possess varying degrees of tolerance towards desiccation (both gradual and rapid). In contrast, for slow or gradual desiccation, species of the two genera (*Steinernema* and *Heterorhabditis*) can manifest enhanced survival reflected by morphological, biochemical, and molecular adaptations.

Having indicated the molecular bases of desiccation/heat tolerance, it is noteworthy to mention remarkable examples. The selection of nematodes that can withstand the desiccation process has successfully produced strains of EPNs that can survive this process with a high rate of survival [86]. Moreover, EPN heat tolerance has also strongly flexible genetic components. This trait was shown to be improved in individuals that were allowed time to adapt to dry environments too [26]. Hence, desiccation and heat tolerance traits can be improved via selective breading so that the EPN lines can withstand a broader/higher range of temperatures. Glazer and Shapiro-Ilan [12] reported the ability to transfer heat tolerance between two strains of *H*. *bacteriophora*. Transfer of this trait was accomplished by mating the heat-tolerant strain IS-5 with the laboratory strain HP88. The hybrid nature of the progeny was affirmed with a mutant of the HP88 strain (H dpg-2) as a marker and via backcrossing. Progenies from the cross were screened for heat tolerance by measuring survival after 2 h of exposure to 40 °C. After six passages through last-instar larvae of *G. mellonella*, survival of the hybrid nematodes was significantly greater than that of strain HP88 and similar to that of strain IS-5. Interestingly, both strains IS-5 and the hybrid killed *G*. *mellonella* larvae at a faster rate than strain HP88 at 32 °C. Conversely, both strains IS-5 and the hybrid showed sensitivity to cold storage at 10 °C, but no differences were detected in reproductive potential. Such results documented that the heritability of heat tolerance is relatively high for some species like *H*. *bacteriophora* but lower in other EPN species [76]. Moreover, within the negative context of trade-offs, other issues may appear. Inbreeding can have depressive effects on many desired traits. Some characteristics that are most highly affected include heat tolerance and fecundity [26]. Nonetheless, trait deterioration can be defeated via outbreeding. Outbreeding uses an outside line of EPNs that is mixed with the existing lines for breeding, and this method has demonstrated success at restoring trait deterioration due to inbreeding [87]. Ultimately, EPN performance under interacting environmental pressures could be strengthened, as shown in the recent comparative literature [88], which emphasizes that biological outcomes often emerge from interactions among multiple biotic and abiotic stressors rather than from single stressors in isolation.

***Co-optimization of EPNs and their symbionts****:* The commercial success and biocontrol efficacy of *Steinernema* spp. and *Heterorhabditis* spp. rely completely on their obligate mutualism with their partner species of *Xenorhabdus* and *Photorhabdus*, respectively. So, co-improving this bipartite system requires an integrated understanding of the evolutionary, physiological, and technological forces that govern both partners. Upon entering an insect, the IJs regurgitate these symbionts found in definite portions of their bodies into the host’s hemocoel to initiate infection. As the pairing between EPNs and their bacteria is highly specific, artificial pairing of an EPN with a non-native symbiont strain to achieve higher virulence often results in a severe drop in reproductive fitness or fails to package the bacteria into the next generation of IJs [3,9]. So, compatibility of the dyad must be assured in any programs for EPN improvement. Another hurdle in co-optimizing the symbiosis is bacterial phase variation, where the bacteria spontaneously switch between two morphologically and physiologically distinct states, that is, Phase One Variants and Phase Two Variants. Managing or genetically stabilizing the bacteria in Phase One during programs for EPN improvement is critical to keeping EPN–bacterial co-cultures highly productive. Inside the host hemolymph, the EPN–bacterial complex acts as a coordinated pathogenic unit to overcome the insect’s immune system. The symbionts secrete an arsenal of high-molecular-weight insecticidal toxins to suppress hemocyte-mediated phagocytosis, induce apoptosis in immune cells, and cause fatal toxemia or septicemia often within 48 h. In turn, EPNs actively secrete immunomodulatory/venom proteins that contribute to attaining insect mortality [3]. Surely, over-expressing such symbiont/EPN compounds for superior suppression of insect immunity in breeding programs can improve the virulence of the dyad. On the other hand, in vitro liquid fermentation bioreactors can largely scale up EPNs, but they rely on a synthetic environment. This latter introduces mechanical and biological stressors that can disrupt the EPN–bacterial mutualism. Therefore, specific factors should be followed, e.g., monoxenic culture maintenance and avoiding shear stress and desynchronization during the programs [23]. Finally, breeding programs designed to optimize specific EPN biocontrol traits frequently trigger negative, unintended evolutionary trade-offs between nematode and bacterial traits [12]. A striking example is that nematodes (IJs) carrying more symbiotic bacteria are more effective at parasitizing insects. Yet, these IJs suffer shorter lifespans in the soil if they cannot find a host quickly compared to IJs carrying fewer symbionts [89]. Hence, co-optimization strategies must balance these competing traits, ensuring that selective improvements to one partner do not undermine the overall fitness and performance of the mutualistic symbiosis.

### 2.4. Techniques That Directly Target the EPN Genome for Biocontrol Improvement in Omics Era

Although some scientists consider the pre-omics era to generally be the period before the 1990s in biological research [90], it is difficult to ignore the disparity between countries in keeping pace with modern genomics. With such overlaps between the pre-omics and omics eras, the latter can be characterized by the emergence of large-scale, high-throughput global analyses of biological molecules (such as genomics and proteomics), especially in developed countries. Therefore, researchers aspire to investigate complete genome sequences via addressing genome-wide analyses of EPNs [91] and to exploit automated bioinformatics databases to map cellular contents globally [8]. Instead of investigating single genes, proteins, or metabolic pathways one at a time, molecular biologists are looking at entire systems of EPNs [92]. Eventually, from the above-mentioned molecular tools commonly used in the pre-omics era, a clear shift is progressing from isolated molecule observation to a holistic, systems-biology approach analyzing all cellular components simultaneously at the omics era. Striking examples of experiments and attempts related to improving EPN applications in the omics era include the following approaches that can physically alter the DNA structure of the genome.

#### 2.4.1. Mutagenesis Approach

Approaches like *mutagenesis* and RNAi were mentioned earlier for improving the EPN “*Infectivity*” trait, but more relevant explorations of additional ways are needed to enhance other biocontrol attributes. Simply, modern genetic and molecular techniques are ongoing to enhance any part, not a single one, of the cascade leading to superior EPN biocontrol tactics and strategies. As molecular genetics specifically uses molecular tools to study genes, related mutagenesis and genetic engineering approaches are being heavily involved in improving such biocontrol traits of EPNs. The induction of mutations should represent remarkable tools for EPN genetic analysis and improvement. It has the privilege of stretching the improvement program beyond the limits of the natural gene pool. Glazer and Shapiro-Ilan [12] recorded that the bulk of mutagenesis and genetic engineering investigations have focused on *Heterorhabditis* spp. more so than on *Steinernema* spp. Zioni et al. [93] used a homozygous inbred line of *H*. *bacteriophora* as the base population for mutagenesis and genetic analysis. Mutagenesis was induced and characterized by exposing young hermaphrodites to 0.05 ethyl methansulfonate (EMS), and a dumpy (dpy) mutant (*Hdpy*-*1*) was isolated. Morphological analysis showed distortion of the head region in adults as well as in IJs. Backcrosses with the wild-type population and genetic analysis indicated that the mutation is recessive. Later on, more recessive dpy mutants (*Hdpy*-*2* and *Hdpy*-*3*) were isolated and characterized. Complementation tests indicated that each of the three mutations affects different genes [12]. Importantly, the *Hdpy*-*2* mutant was used as a genetic marker to validate crosses between heat-tolerant and heat-sensitive strains [24].

In contrast, Abd-Elgawad [8] stressed that transposon mutagenesis has been more dependable than chemical mutagenesis, e.g., using EMS. That is because transposition mutagenesis possesses a higher frequency of mutations and lower lethal efficacy. Moreover, he reported that the resulting type of mutagenesis can incorporate chosen markers in EPN strain construction, produce single-hit mutations, and recover genes after mutagenesis. The disadvantages of it involve its low frequency in living systems and imprecision with most related systems. Two novel *H*. *bacteriophora* mutants with evident morphological characteristics acted as genetic markers for indicating the desired trait(s) or for mapping of functional genes [94]. As new EPN genes are discovered, mutant rescue phenotype analysis can be a beneficial approach for identifying genes. In a different study, mutagenesis screens took advantage of the genetically tractable system of *S*. *hermaphroditum* to identify its features of reproduction [95]. This approach used *S*. *hermaphroditum* as a genetic development model to investigate both insect parasitism and the related naturally occurring symbionts. The authors [95] utilized linkage mapping and complementation testing to localize these mutations, laying the groundwork for subsequent methods of gene knockouts.

#### 2.4.2. Genetic Engineering Approach

There are no recent breakthroughs or active reports of stable genetic transformation (transgenesis) for EPNs in the current publications. Yet, encouraging examples of successful genetic transformation of EPNs were recorded in the recent past [96,97]. Progress had been made in using transgenic approaches to enhance EPN efficacy [98,99]. However, genetically modified EPNs have not yet been commercialized, nor have researchers in the field developed these methods further. In contrast, there are now multiple ways to develop transgenic strains of nematodes, such as microinjection of genetic constructs into the gonad and microparticle bombardment [26]. Although microinjection has been used to make transgenic EPNs [26], this technique has not been adopted further. Several practical limitations are faced and should be resolved in the cascade of improving EPN traits. The apparent limitation of microinjection is that the EPN’s resilient cuticle and high internal hydrostatic pressure could make inserting a needle without bursting or killing the nematode exceptionally difficult. Baiocchi et al. [26] presumed that microparticle bombardment, which is carried out on many other transgenic nematodes and with multiple genetic constructs at the same time, will therefore have more success in the future of EPN communities. Also, achieving stable, non-mosaic integration into the genome, e.g., via CRISPR-mediated knock-in or transposon systems, remains inefficient in non-model nematodes. So, molecular biologists often have to work with transient or heavily mosaic lines. Likewise, securing germline transmission from a single injected, surviving founder requires successful mating (in amphimictic species, mostly of *Steinernema* but not hermaphroditic *Heterorhabditis*) and successful transition into IJs. If the transgene disrupts fitness or the nematode’s ability to host its symbiotic bacteria, the line will fail to transition through generations. Also, EPN genomes often suffer from low completeness, high fragmentation, and poor annotation due to repetitive elements and high heterozygosity, which limits availability and degrade the quality of the intended reference genomes. On the other hand, unintended genetic modifications can occur at non-targeted genomic regions that share high similarity with the guide RNA. Evaluating these errors in EPNs is highly challenging because a lack of comprehensive reference genomic data means standard computational models cannot easily predict or spot these unintended cuts, resulting in off-target evaluation [46,52,53,67]. Likewise, precision gene editing depends on the homology-directed repair (HDR) pathway to introduce specific elite alleles or custom templates. However, the default cellular mechanism in nematodes favors the error-prone *Non-Homologous End Joining* (NHEJ) pathway, resulting in low knock-in success rates and high rates of unwanted insertions or deletions [100]. Furthermore, EPNs have complex life cycles, usually engaging obligate dependencies on their symbiotic bacteria and complex mating habits. These habits are especially important for many commercial *Steinernema* species, as they require separate males and females. In this sense, breeding, maintaining homozygous edited lines, and preventing genetic drift or strain degradation during long-term lab colonization are highly difficult, often leading to the unstable maintenance of improved strains, as mentioned previously [bioRXiv]. Finally, modifying a single gene to improve an isolated biocontrol trait (e.g., maximizing heat resilience or environmental persistence) can inadvertently damage other critical biological traits, such as host-seeking behaviors, reproductive fitness, or mutualistic compatibility with their bacterial symbionts, via pleiotropic effects [12,101].

This does not negate the fact that introducing exogenous or modified genes into EPNs in a consistent and reliable manner is paramount to our ability to employ molecular methods for genetic improvement of EPNs in biocontrol. The huge amount of knowledge, tools, and techniques developed for EPN research can be harnessed to further improve them. This is especially important because EPN genetic transformation can materialize improvements that are not possible with traditional techniques for specific beneficial traits. Therefore, various approaches should be anticipated and developed [26]. Within this context, inserting more copies of beneficial genes to boost the already present ones is an existing possibility that has been achieved with other BCAs. St Leger et al. [102] constructed an improved myco-insecticide over-expressing a toxic protease. They transformed multiple endogenous cuticle-degrading proteases (Pr1) into the genome of the entomopathogenic fungi *Metarhizium anisopliae* (Metchnikoff) Sorokin (Hypocreales: Clavicipitaceae) such that Pr1 was constitutively over-generated in the hemolymph of *Manduca sexta* L. (Lepidoptera: Sphingidae), activating the prophenoloxidase system. Another way of offering a beneficial genotype was achieved via adding novel secreted bioactive compounds, such as those encoded by a scorpion venom gene, into the *M*. *anisopliae* genome [103]. It elevated its pathogenicity against coffee berry borer, *Hypothenemus hampei* Ferrari (Coleoptera: Curculionidae), as *M*. *anisopliae* expressed the scorpion toxin (*AaIT*) gene. This approach may enforce *H*. *bacteriophora* pathogenicity which has fairly few predicted proteases and protease inhibitors in its secretome. Given such a status, *H*. *bacteriophora* depends on its symbiotic bacteria for immune suppression of its insect host [26]. Hence, inserting more copies of genes in charge of EPN-secreted proteases can have more influence on nematode penetration into the insect hemolymph [92], immune suppression [103], and degradation of insect tissues [104,105]. Obviously, such a transformation of superb genes from fungi or bacteria to EPN species does not mean that the genetic constitution of definite EPN species/strains is empty from other superior genes [78]. This is materialized and exemplified by a unique heat-stable, water-stress-related protein, named DESC47. It is encoded by the desc-47 gene, which is present in the *S*. *feltiae* strain IS-6 [106]. Also, Wang and Gaugler [107] reported EPN surface coat proteins in *S*. *glaseri*. They found that at least one protein (SCP3a), when injected into an insect host, defends unrelated EPN species against encapsulation and latex beads against phagocytosis. Therefore, obtaining EPN strains/lines with promising biocontrol attributes via genetic improvements of a sole EPN gene pool should be an ongoing process. Nonetheless, the aforementioned extrapolation using BCAs other than EPNs is still considered reasonable by some nematologists [8,26]. They have presented it as a model to be emulated for improving nematode performance too. The end in view is to develop upgraded EPNs so that they can be effectively and inexpensively utilized as bioinsecticides worldwide [73,74,75,76,77,78]. Meanwhile, care must be taken to address the obstacles facing EPN genetic improvement so that some obtained traits do not negatively affect other desirable traits [12]. Eventually, further studies are warranted to substantiate these improvements and offer the best guidance to stakeholders.

#### 2.4.3. Using the Clustered Regularly Interspaced Short Palindromic Repeat (CRISPR) Tool

Further techniques may be utilized for genome editing schemes to develop promising strains [8]. The relevant perception is that such schemes for genetic enhancement should be scalable with more EPN products to affirm their advantages under field settings. In this respect, O’Halloran [108] set computer software called CRISPR-PN2 (CRISPR-Parasitic Nematodes version 2) as an adaptable and genome-aware platform to aid in genetic tests on parasitic nematodes. It offers resilient usage and control over the automated sketch of specific guide-RNA sequences for CRISPR experimentations on PPNs. CRISPR-PN2 is a computational guide-RNA design platform for parasitic nematodes, not itself evidence that genome editing has been experimentally achieved in EPNs. In other words, its program helps in enabling high-throughput gene editing at the desired scale. Clearly, such editing may help in supporting desired traits in plants that do not have enough resistance sources against pests. A modified CRISPR-Cas9 tool has associated protein 9 (Cas9). This protein—Cas9—acted as an enzyme that cuts DNA strands and relied on a guide-RNA molecule to locate the exact sequence to cut for genome editing in two EPN species, *S. carpocapsae* and *S. hermaphroditum* [109]. This study proved that species of *Steinernema* are highly tractable for targeted mutagenesis, with potential investigations of gene functions under controlled conditions.

A particularly relevant demonstration of functional genomics editing in an EPN (*S. hermaphroditum*) could combine three components—whole-genome resequencing (WGS), genetic mapping, and CRISPR-Cas9—to mark, for the first time, a mutationally defined phenotypic locus at the molecular level [53,95]. Each of these components offered specific benefits. Genetic mapping served as the initial “GPS,” pointing researchers to the approximate physical and chromosomal location of the mutation causing an altered body size and shape. So, it successfully determined that the *skpo-2* locus is X-linked (for chromosomal localization). Another benefit of genetic mapping is that it narrowed the search space. By examining recombination and inheritance patterns in *S. Hermaphroditum* (which is uniquely advantageous because it reproduces by self-fertilizing hermaphrodites), mapping was sharply restricted to the candidate genomic region. This saved significant computational and sequencing resources that would have otherwise been wasted analyzing the entire 90.7 Mb genome. Following this narrowing, WGS functioned as the high-resolution microscope to find the exact nucleotide alterations. So, resequencing EMS-mutated strains allowed the authors to find the specific base-pair changes within the mapped region. It revealed that the phenotype was caused by disrupting an ortholog of the essential *C*. *elegans* peroxidase gene, *skpo-2*. So, WGS offered an unexpected secondary benefit: it allowed them to profile and understand the novel mutational spectrum of EMS mutagenesis unique to *S. hermaphroditum*, establishing baseline data for future forward-genetic screens. Identifying a candidate gene via WGS and mapping is only correlative; CRISPR-Cas9 was required to definitively prove causality (direct causation proof). By using CRISPR-Cas9 to engineer targeted, independent loss-of-function knockouts in wild-type *S. hermaphroditum*, the authors perfectly recreated the “Dumpy” body size and shape phenotype. This conclusively validated that *skpo-2* was the correct gene (definitive functional confirmation). In *C. elegans*, *skpo-2* is an essential gene, making it difficult to study. CRISPR-Cas9 allowed the authors [53] to safely target and dissect its non-lethal, morphological role in *S. Hermaphroditum* (bypassing lethality constraints). Because *S. hermaphroditum* reproduces primarily through self-fertilization, any targeted edits were made via CRISPR quickly and easily transitioned into genetically stable, homozygous mutant lines without the need for complex cross-mating schemes (leveraging hermaphroditic genetics). Factually, validating this specific locus, via this toolkit to uncover *skpo-2,* opened new avenues of key tools for further EPN research.

## 3. Other Sophisticated EPN Molecular Techniques

Developments of such techniques are ongoing, with advantageous outputs for enhancing EPNs’ ability as BCAs and/or reducing costs, the two main components that are hindering their wider participation in the pesticide market.

### 3.1. Using RNAi to Study Gene Function or Silence Targeted Genes

As aforementioned, advanced molecular tools like RNAi and sequencing will continue to provide new avenues for improved efficacy as more biocontrol genes are identified [12,82]. Since RNAi can determine the function of a gene, it can be tailored to target a specific gene of interest, silence the gene in vivo, and observe the effect this has on the EPNs. It works downstream at the transcript level without changing the underlying genomic sequence. Thus, RNAi may serve as a versatile platform, with contributions demonstrating its dual role as a crucial research methodology and an influential tool for pest control. In a study by Morris [109], the *flp-21* gene was suppressed by 84% (*p* < 0.0001) by soaking *S*. *carpocapsae* IJs in a high amount of double-stranded RNA (dsRNA) for 48 h with 50 mM serotonin. The suppression of *Sc-flp-21* had significant effects on the host-finding behaviors via impeding olfactory perception in the nematodes. The study used another method, i.e., RNA sequencing, to identify genes that regulate intraspecific phenotypic differences between populations. Sequencing the transcriptome permitted observation of the gene expression pattern of a single cell or entire organism at one time. Differentially expressed genes between phenotypically different populations showed the genes responsible for the intraspecific differences noted between populations. Hence, transcriptome analysis identified differentially expressed genes between *S. carpocapsae* strains that showed phenotypic differences with regard to their host-finding behaviors [110]. On the positive side of this study, gene knockdown had successfully silenced target transcripts in IJs. Also, it attained behavioral modification because focusing on neuropeptide-like protein genes could alter movement, ecological responsiveness, and host-seeking performance. Moreover, transcriptomic integration via combining RNA- sequencing with RNAi led to mapping out the functional pathways responsible for intraspecific phenotypic variations. On the negative side, there are delivery obstacles in moving the controlled in vitro soaking method of EPNs to reliable field applications. Therefore, RNAi was also applied successfully in *H. bacteriophora* [110], but it has not been adopted by the field at large. Also, an EPN efficacy gap is usually found between laboratory and field conditions. Overcoming this issue requires careful study of field settings to unlock molecular triggers that govern EPN–host interactions to improve the nematode effectiveness.

### 3.2. Combined Phenotypic and Genotypic Characterization for Sound Molecular Markers

Combining the degree of tolerance to heat and desiccation stress (phenotypic level), with variations in gene expression and genome sequence (genotypic level), was exemplified by Levy et al. [111]. They examined the osmotic desiccation tolerance via exposing natural populations of *H*. *bacteriophora* IJs to polyethylene glycol 600. After this osmotic desiccation stress, genomic variation and gene expression patterns were inspected for genetic diversity. This transcriptomic study enabled the identification of genes and molecular markers that can be used as genetic selection tools to upgrade EPN tolerance to ecological extremes. To raise the EPNs’ environmental stress resistance as a precedence for their biocontrol effectiveness, more than 80 *H*. *bacteriophora* wild-type (WT) strains and inbred lines were fully screened for their IJ longevity and EMS mutants. Their extended survival was bred via selection. Thereafter, genotypic and phenotypic data were integrated to define genes, DNA polymorphisms, and genotypes with superior potential for improvement [16]. Thus, merging desirable EPN traits by using high-throughput genotyping screen with EPN breeding is quite possible for their effective biocontrol.

### 3.3. Extensive Breeding and Selection to Reduce Costs of EPN Application

Field experimentations utilizing standard farming machinery were carried out in different European countries to suppress populations of the Western corn rootworm (*Diabrotica virgifera virgifera* LeConte [Coleoptera: Chrysomelidae]) by superior selection of marketable *H*. *bacteriophora* IJs, applied at 2 × 10^9^ ha^−1^ with 200 L ha^−1^ of water into the field furrows together with the maize seeds. The *H*. *bacteriophora* IJs caused a 65% decrease in the pest population level. This reduction surpassed outputs gained by the chemical standards in 11 of the 16 trials [22]. Such a remarkable example utilized a more persistent and virulent *H*. *bacteriophora* line from genetic breeding. Notably, it justified a decrease in the EPN application rate to one-half that of the commercial nematodes. Hence, it has the advantage of minimizing costs to the range of synthetic chemicals [22]. Similarly, new hybrid inbred lines of commercial *H*. *bacteriophora* were selected for optimized traits for better mass production, longer persistence, and higher virulence in another study [112]. Two strains (promising H2L3 strain from the breeding program and commercial EN01 strain) of this EPN species were compared to a standard granular soil insecticide (the pyrethroid cypermethrin) in two heavily *D*. *v*. *virgifera*-infested maize fields [112]. The two strains were more effective than cypermethrin (16%). Such extensive breeding and selection operations for the desired traits in the related programs may usually imply molecular processes like differential gene expression, olfaction-associated signal transduction, stress-tolerance pathways (e.g., trehalose synthesis), and symbiotic bacterial maintenance to enhance EPN effectiveness in the field. Interestingly, these processes can track changes in traits like other environmental stresses and host cue responsiveness [12,73,75].

### 3.4. Using EPN Whole Genomes with Other Tools for Taxonomy and Possible Editing

Emerging molecular tools will continue to provide new avenues for improved EPN efficacy as more biocontrol genes are identified. A superb model of superior attributes for genetic improvement in the biocontrol of insect pests has recently been nominated by Ehlers et al. [113] as *H*. *bacteriophora*. To this end, advanced technologies should be adapted for EPN genetic application to integrate novel genomic, bioinformatics, and proteomic expertise. The genome of this species (TT01 strain) has already been sequenced [92]. This genome size of ~77 megabase pairs (Mbp) is based on a draft genome sequence of *H*. *bacteriophora,* which highlights its unique evolutionary position. It bridges free-living nematode models like *C*. *elegans* and vertebrate parasites, alongside its mutualism with the pathogenic bacterium *Photorhabdus luminescens*. Recently, whole nuclear and mitochondrial genome sequences have rightly resolved phylogenetic relationships of the genus *Heterorhabditis*, avoiding conflicting tree topologies that are often procured via different gene markers [91]. In their nuclear and mitochondrial genome sequencing and assembly procedures, they used bioinformatics tools for high-quality reads before reconstructing both the whole nuclear and mitochondrial phylogenomic level and whole ribosomal RNA operon and single-gene phylogenetic level. So, they set co-phylogenetic relationships and biogeography patterns using the “phylo.to.map” function. In contrast, if whole nuclear and/or mitochondrial sets are not obtainable, nuclear and mitochondrial genes, especially the cytochrome c oxidase subunit I (cox-1), the NADH dehydrogenase subunit 4 (nad-4), the fanconi-associated nuclease 1 (fan-1), and the serine/threonine-protein phosphatase 4 regulatory subunit 1 (ppfr-1), can be used together instead. Yet, in such cases, genus-wide analyses of such genes, involving multiple conspecific isolates, are highly recommended to evade artifactual species over-splitting and species lumping [91]. Ultimately, having made such progress, there is a need to expand high-throughput sequencing of EPN genomes along with molecular markers to facilitate the tracking of desired traits as well.

### 3.5. High-Throughput Sequencing (HTS) Metabarcoding and qPCR Yield

Metabarcoding outputs can enable more comprehensive and fine-scale characterizations of EPNs. Its related identification and characterization of EPNs outperformed other methods used in former surveys to detect undescribed EPN species, yet the optimal method depends on the goal [54]. These authors discovered DNA sequences that have not been recorded in the GenBank archives. Moreover, metabarcoding offered fine-scale taxonomic resolution, enabling precise distinction of closely related EPN species. This differentiation is important because these species can manifest distinct phenotypes of key traits that determine the best EPN–host matching. Examples of such traits involve insect habitat adaptation, persistence, host specificity, and reproductive potential. Additionally, the metabarcoding technique discovered more EPN species than did qPCR. Yet, the impacts of biotic/abiotic factors on each of these EPN species were not substantially different between the two molecular methods (qPCR and HTS) [54]. In another study [114], the impacts of pest management (integrated vs. organic), soil management (tilling vs. cover cropping), and their interplay on feeding type fractions involving the climatic zone (Winkler index) as a random element were evaluated. This study used HTS metabarcoding to identify 114 nematode taxa in order to appraise trophic composition, nematode diversity, and network connectivity across 57 vineyards. The results indicated that organic viticulture considerably elevated nematode diversity (*p* < 0.001), food web complexity, and ecological stability, as reflected in meaningfully higher (*p* < 0.01) Maturity Index and Structure Index values, along with lower (*p* < 0.01) Basal Index values. Also, organic vineyards showed greater network connectivity, demonstrating a more structured and resilient soil food web than integrated pest management (IPM). On the contrary, cover cropping and tillage had limited impacts on nematode community structure and soil health indices, showing a weaker effect on nematode-mediated soil functions in these settings. The authors concluded that organic viticulture is quite useful in raising soil biodiversity and ecological stability, fortifying the role of sustainable farming exercises in enriching soil health [114]. Dritsoulas et al. [115] pointed that metabarcoding can provide a more cost-effective and authentic means of examining soil food webs than other currently available methods like qPCR. They found that computing both HTS and qPCR data to fit Taylor’s Power Law demonstrated that sample accuracy was substantially better using HTS (r^2^ = 0.95, *p* < 0.01) than qPCR (r^2^ = 0.76, *p* < 0.01). Thus, it is apparent that merging metagenomic and genomic insights is promising for further durable agriculture.

In contrast, because abiotic and biotic factors can influence EPN biocontrol potential, Campos-Herrera et al. [116] reliably used qPCR to detect and measure organisms at different trophic levels to compare a new citriculture system (NCS) that fertigated citrus trees daily (to combat citrus greening disease) with conventional citriculture (CC) in Florida citrus orchards. The target organisms comprised six EPN species, five nematophagous fungi (NF), and a phoretic bacterium, *Paenibacillus* sp. Ash et al. (Caryophanales: Paenibacillaceae). Soil traits, free-living nematodes, and citrus fibrous roots were also measured. They found that NCS raised soil pH and Mg content, decreased the electrical conductivity, and decreased the contents of K, Mn and Fe with consequent suppression of native and augmented EPNs. Moreover, the naturally occurring *S*. *diaprepesi* Nguyen & Duncan (Rhabditida: Steinernematidae) was 5 times less abundant in plots of citrus trees where these nematodes were more heavily encumbered by the phoretic bacterium *Paenibacillus* sp., which limits the foraging and biocontrol success of EPNs. This context-dependent nature means a beneficial microbe (e.g., *Paenibacillus* sp.) in one environment may be harmful or at least not beneficial in another. Thus, the holistic approach that involved qPCR as an effective tool considers diverse benefits of these microorganisms but under specific settings. Wu et al. [117] completed a spatial analysis by distance indices (SADIE) program and also applied qPCR to discover significant spatial links between the fungus *Fusarium solani* (Mart.) Sacc. (Hypocreales: Nectriaceae) and polyspecific EPN communities, *S. diaprepesi*, *H. indica*, and *H. zealandica* Poinar (Rhabditida: Heterorhabditidae), during a 6-month citrus grove survey. The EPNs had aggregated distributions during three continuous winter months, but the fungus showed clumped distribution during the previous month. This pattern did not exist during the spring. The *F. solani* aggregates in the surveyed plots were linked to that of the total nematodes the previous month, with a one-month time lag as well (r = 0.17, *p* = 0.02). The authors presumed that such a pattern may be at least partly due to the impact of soil moisture on both EPNs and *F. solani*. The demonstrated relationships between *F. solani* and EPNs clearly disclosed a field relationship between both organisms. Obviously, a weak lagged association between EPNs and *F. solani* should not be regarded as definitive evidence of field mutualism.

Finally, these molecular data combined with indices of dispersion should be compared to the related biotic and abiotic forces to unravel the complexities of ecosystem dynamics into quantifiable variables for better development of pest control [118].

As EPN inundative release is the mostly used technique, EPN populations often diminish to low levels within a few post-application weeks, representing a real issue. To track and understand the causes of this issue, research is being conducted in integrated directions. A former study [119] developed qPCR assays that detected and quantified six EPN species, naturally distributed in Florida citrus orchards. The authors optimized a protocol for extracting nematodes and DNA from soil samples that can detect only one EPN added to nematode communities. This and similar related protocols could quantify soil-inhabiting organisms such as EPNs and their relevant natural enemies and competitors of free-living nematodes, ectoparasitic bacteria, and nematophagous fungi in the intended soil. Also, they can examine the EPN soil food web in the studied fields. Another merit of qPCR as a relevant molecular method was its use to disclose sympatric distributions of EPN species and reveal their low levels in samples, but the insect baiting technique could not, that is, an EPN detection method using a molecular vs. traditional approach [118,119,120]. Such tools should be harnessed to track and grasp low EPN levels within a few post-application weeks in the targeted settings to enhance the EPN common inundative strategy.

A reverse transcription quantitative PCR (RT-qPCR) process was employed as effective method to measure gene expression. It can independently confirm, measure, and validate specific changes in mRNA transcript levels identified through large-scale screening tools like RNA sequencing, verifying differential gene expression (e.g., up- or downregulation), as discovered in transcriptomic studies. Liu et al. [121] determined differentially expressed genes and linked enriched pathways via analyzing the transcriptomic response of *H*. *bacteriophora* strain H06 in the liquid medium with 0.01% dimethyl sulfoxide (DMSO). They examined the operation of the selected genes by employing RNAi. After DMSO treatment, they tracked regulation of relevant bioprocesses, including metabolism, binding, signal transduction, post-transcriptional modifications, metabolism, and protein folding. By using RNAi, the knockdown of three genes, *PAN domain protein*, *Hint module*, and *Dpy-13*, substantially regulated the egg load (at least a 17% gain in eggs per hermaphroditic adult) and the yield of IJs (at least a 48% increase). The results of RT-qPCR [121] confirmed that the expression levels of the knockdown genes decreased significantly, relative to the control. Their results represent a new technology for raising nematode commercial production.

## 4. From Molecular Mechanisms to Ecological Deployment

Having stressed a few examples of a few molecular techniques to illustrate their updated counterparts, it is important to integrate mechanistic and systems-level approaches. This does not negate that comprehensive recognition of the complexes of EPNs and their symbionts can address the mutualistic bacteria separately to improve biocontrol traits of the dyad. Valuable components of their secondary metabolites that are toxic to insects have recently been reviewed [122] to discuss how to exploit their possible role in improving biocontrol of key pests. Molecular docking and molecular dynamics simulations can substantially advance manipulation of these metabolites by enabling precise predictions of ligand–target interactions. Clearly, docking predicts potential interactions, but it does not establish a biological activity or mechanism without experimental validation.

A striking example was given by *Xenorhabdus nematophila* Poinar and Thomas (Enterobacterales: Morganellaceae) symbiotically associated with *S*. *carpocapsae*. A wide screening of *X*. *nematophila* cell-free supernatants (CFSs) utilized gas chromatography–mass spectrometry and identified 61 metabolites in the CFSs. Thereafter, docking simulations showed strong ligand binding affinity to pest (*Tetranychus urticae* Koch [Trombidiformes: Tetranychidae])-specific proteins; that is, 2-octyl-1H-quinolin-4-one indicated robust binding to *T. urticae* proteins that comprise TuAchE (−8.8 kcal/mol), TuGluCl (−6.1 kcal/mol), and TuMETI (−7.7 kcal/mol). The strong binding indicated potential disruption of essential physiological functions in the mite, *T. urticae* [123]. It is well known that such ligand–target interactions can accelerate virtual screening and optimize lead compounds [124]. The authors stressed that using these techniques, the mechanism of action would also be predicted efficiently without being time-consuming but with minimal resources relative to the traditional methods. Hence, accurate predictions of such ligand–target interactions should be expanded to other EPN-symbiotic bacterial species to exploit the arsenal of their secondary metabolites against key pests. Furthermore, while a 91.01% reduction in the *T. urticae* population on lablab plants 14 days after CFS application was achieved, minimal toxicity of these CFSs to beneficial organisms like honey bees and predatory mites was recorded [123]. Anticipated possibilities of such bacterial components are to boost insecticidal virulence, bypass host immune defense, increase ecological fitness, and stabilize mutualistic interactions. Yet, since docking affinity alone does not establish a biological target or insecticidal mechanism, computational predictions require orthogonal validation. Also, systems-level and mechanistic approaches should be exploited as complementary, not mutually exclusive. Multi-omics and ecological network analyses are most informative when coupled to causal experiments, while mechanistic results gain relevance when tested in realistic ecological systems. Moreover, non-molecular techniques should be incorporated into molecular ones, if applicable, for extensive and reliable biocontrol via the dyad (EPNs and their symbionts) or the symbionts alone.

Comprehensive techniques were efficiently deemed to compare traditional versus recently introduced agricultural practices. Blanco-Pérez et al. [125] used classical (insect baits) and molecular (qPCR analysis) techniques to assess the abundance and activity of native EPNs and the existence of natural enemies in vineyard soils of Spain. They found that organic mulches favored biotic conditions for EPNs compared to herbicide application or tillage. They concluded that the organic mulches can offer proper conditions for raising nematofauna numbers but, depending on the mulch type and the abundance of IJs related to EPN natural enemies, may also negatively affect EPNs by elevating their enemies. Interestingly, six field experiments (in two Egyptian governorates for three strawberry seasons) showed that combining EPNs with mulching improved the EPN field efficacy against white grubs *Temnorhynchus baal* Reiche & Saulcy (Coleoptera: Scarabaeidae) relative to the non-mulched strawberry beds [126]. The authors stressed that related biotic and abiotic factors should also be considered for optimizing EPN effectiveness under various conditions. On the other hand, Manduca et al. [127] introduced an interdisciplinary technology to examine the behavior of *S. carpocapsae*. They employed a novel blend of deep learning and optical flow analysis, and a Convolutional Neural Network effectively recognized how *S. carpocapsae* reacted to host-borne stimuli. Their model demonstrated EPN behaviors in a prototyped microfluidic platform reproducing a host-environment context with high precision and an overall accuracy of 0.938. Their integration of optical flow analysis highlighted an enhanced motor activity in EPNs when exposed to stimuli, adding novel information on their dynamic responses. Their methodology offers a significant progress in detecting and understanding EPN responses to diverse stimuli, fostering their use as advanced BCAs in sustainable pest control and environmental management.

To sum up, remarkable examples of common molecular methods with their pros and cons are presented along with their different validation levels (Table 1). For example, “ITS” regions for the improvement, laboratory breeding, or characterization of EPNs fall under internal validation, whereas verifying these traits across independent environments, fields, or wild populations represents external validation. Another example falls within the context of evaluating and selecting EPNs for genetic improvement, e.g., breeding/engineering them for enhanced heat tolerance, virulence, or host-seeking traits. In such cases, DNA sequencing falls under both internal and external validation categories depending on how the data are applied.

These methods help researchers to pick the best one for specific pest control settings. Along with the above-mentioned explanations on their merits/demerits, stakeholders may balance the high precision and speed of a particular method against its high cost and/or technical hurdles; e.g., characterization of heterogenicity versus homogenicity of an EPN population. They may also help to evade potential pitfalls in a future relevant methodology that targets a mechanistic rather than comprehensive system. Moreover, specific points and elements processed so far to advance genetic enhancement of EPNs are illustrated in Figure 2. It indicates, for example, that EPN improvements are not limited to natural EPN populations extracted from the same country/locality. Conversely, EPNs introduced from another country may also require an improvement even when used in the classical biological control method. *Steinernema scapterisci* Nguyen & Smart (Rhabditida: Steinernematidae) was detected in Uruguay and introduced into the USA for classical biocontrol of all mole cricket species that were susceptible to its infection. Nguyen and Smart [128] selected an *S. scapterisci* isolate that, in initial pathogenicity tests in Petri dishes, caused the highest mortality of two species of mole crickets, *Neoscapteriscus* (Synonym: *Scapteriscus*) *vicinus* Scudder (Orthoptera: Gryllotalpidae) and *N. borellii* (Synonym: *S. acletus*) Giglio-Tos. The virulence of the isolate was increased by serial passage (i.e., repeatedly exposing IJs collected from infected mole crickets to other mole crickets) through the two mole cricket species. These nematodes were used thereafter in all future experiments [128]. Ultimately, it is acceptable that the improvements reach natural/imported populations of EPNs.

Fortunately, genetic studies and selective breeding programs have demonstrated that specific fitness and behavioral traits show moderate-to-high heritability (*h*^2^). The traits that can be actively enhanced through direct selection or hybridization are heat (thermal) tolerance, host-seeking ability, desiccation tolerance, virulence, and infectivity [8,9,26]. Also, several methods could be used to maintain trait stability during mass production. These are utilization of homozygous inbred lines (as inbreeding eliminates genetic variation within the line, fixing the desired alleles like heat tolerance), genetic hybridization and cross-breeding (crossing a highly virulent local strain with an exotic heat-tolerant strain creates hybrid progeny that inherit both stable beneficial traits), and monoxenic liquid culture optimization with companion cryopreservation. The latter method strictly offers monoxenic systems (the nematode cultured exclusively with its primary symbiotic bacterium in a sterile environment) while the improved base strain is kept in cryopreserved storage to avoid trait degradation. On the other hand, as *Heterorhabditis* spp. are hermaphroditic in their first generation inside a host (or in media), homozygous inbred line creation via liquid mass culture can easily fix and stabilize recessive traits like heat or desiccation tolerance during automated, monoxenic liquid fermentation [95]. Also, because *Heterorhabditis* can self-fertilize, screening for induced recessive mutations (such as chemical mutagenesis) is highly streamlined. Thus, a mutation is rendered homozygous in just one generation via classical mutagenesis and recessive trait selection. In contrast, almost all commercially relevant *Steinernema* species (like *S. carpocapsae* and *S. feltiae*) are strictly amphimictic across all generations. So, they suffer from severe inbreeding depression when forced into single-pair mating to fix traits. They require cumbersome mass-selection or hybridization programs across populations rather than simple self-mating.

While reverse-genetics tools like CRISPR-Cas9 and RNAi have mapped vital virulence and survival pathways in the laboratory, direct transgenic or CRISPR-engineered nematodes have not yet been released into commercial open fields. However, a few molecular discoveries could be translated to field performance. For the above-mentioned heat-tolerant strains, successful commercialization of *Heterorhabditis bacteriophora*-related strains could significantly extend their active window and survival rate in sun-exposed soils. Other strains with molecular markers in *H*. *bacteriophora* associated with virulence at low temperature are in the pipeline of commercialization [47]. Also, flooding mass-production bioreactors with specific synthetic ascarosides or pre-conditioning IJs prior to spraying alters their post-application behavior. This molecular intervention tricks the nematodes into an active “hunting” state, accelerating soil penetration and boosting field infectivity against target pests [76]. Lastly, quality-control molecular assays now explicitly lock or screen for the Phase One genetic state of symbionts during commercial mass production. Using a Phase One genetic state in mass production could guarantee the best outputs of the culturing. Moreover, it means that the nematodes applied in fields carry highly virulent bacterial strains, preventing the common issue of formulation “attenuation”, where the biopesticide loses its killing power [129].

On the contrary, major knowledge gaps for EPN improvement are still apparent. A critical gap exists in connecting known molecular stress-tolerance mechanisms to commercial formulation of EPNs. For instance, many commercial powder formulations are developed without a full technical understanding of how to artificially induce anhydrobiosis (a dormant state of survival during dehydration). Thus, advancing survival strategies in formulations is needed. Moreover, EPN biology, mass production, and field application should be complementary as integrative research, not separated by researchers. Right from the beginning, the end in view is to achieve superior field application. This would include investigating EPN climatic sensitivity and correct predictability of EPN activities in diverse soil properties. Hence, EPNs in the context of environmental resilience and abiotic/biotic dynamics require further studies. Interactions in the root rhizosphere should be addressed in the given settings on a case-by-case basis to optimize the related biocontrol process and harness the beneficial microbiome [130]. Frequent gaps remain in synchronizing the application timing with other chemical inputs, the precise biology, and behaviors of the target insect pests [5]. On the other hand, researchers have been exploring many novel EPN species and strains, but only a few commercial species are available. So, mass production and scaling of most wild native strains into high-density liquid fermentation systems should be advanced. This gap should be filled up without diminishing their virulence or genetic quality over successive generations. Finally, socio-economic gaps are mostly materialized in EPN costs and a lack of public awareness of the diverse EPN benefits [131].

As a forward-looking roadmap catalyzing future studies over the next years, the EPN research landscape may be perceived as important. It is expected to focus heavily on integrating advanced biotechnology and addressing climate pressures. High priority should be given to molecular and genetic engineering. EPN strains should further be improved via CRISPR/Cas9 and advanced breeding techniques to enhance EPN tolerance to environmental extremes (e.g., heat, desiccation, and UV radiation). Ongoing programs should engineer or select superior strains of Xenorhabdus and Photorhabdus to increase toxin production and virulence, especially against resistant pests. Likewise, sequencing of diverse wild isolates should identify the key genes responsible for foraging behavior, longevity, and host-seeking efficiency. Moreover, developing climate-resilient strains of EPNs is necessary. Hence, the survival, persistence, and subterranean migration of EPNs should be adapted to climate change: rising temperatures and changing soil moisture levels. Therefore, EPNs should be better formulated via developing advanced biopolymer encapsulations, hydrogels, or protective coatings [82]. Such formulations should protect EPNs from temperature, UV light, and desiccation during foliar or shallow soil applications. In field applications, EPNs would be better co-applied with other compatible inputs to secure additive or synergistic control effects in IPM plans. On the other hand, optimizing large-scale, liquid culture efficiency for in vitro bioreactor mass production should be attempted to lower manufacturing costs and increase yields of high-quality IJs. Likewise, molecular tools and techniques should be harnessed for accurate field monitoring of EPNs, their hosts, natural enemies/competitors, and other components in the plant rhizosphere. These involve decoding the specific root-volatile organic compounds that attract or repel EPNs to engineer “push–pull” pest management strategies in the field [132,133]. Also, using devices like qPCR and metabarcoding methods for rapid and accurate detection and quantification of EPN populations and related microorganisms in soil samples post-application will help perfect monitoring. Within this context, utilizing modern and precise systems like “The Smart Soil Organism Detector” should be operationalized and expanded [134].

**Figure 2:**
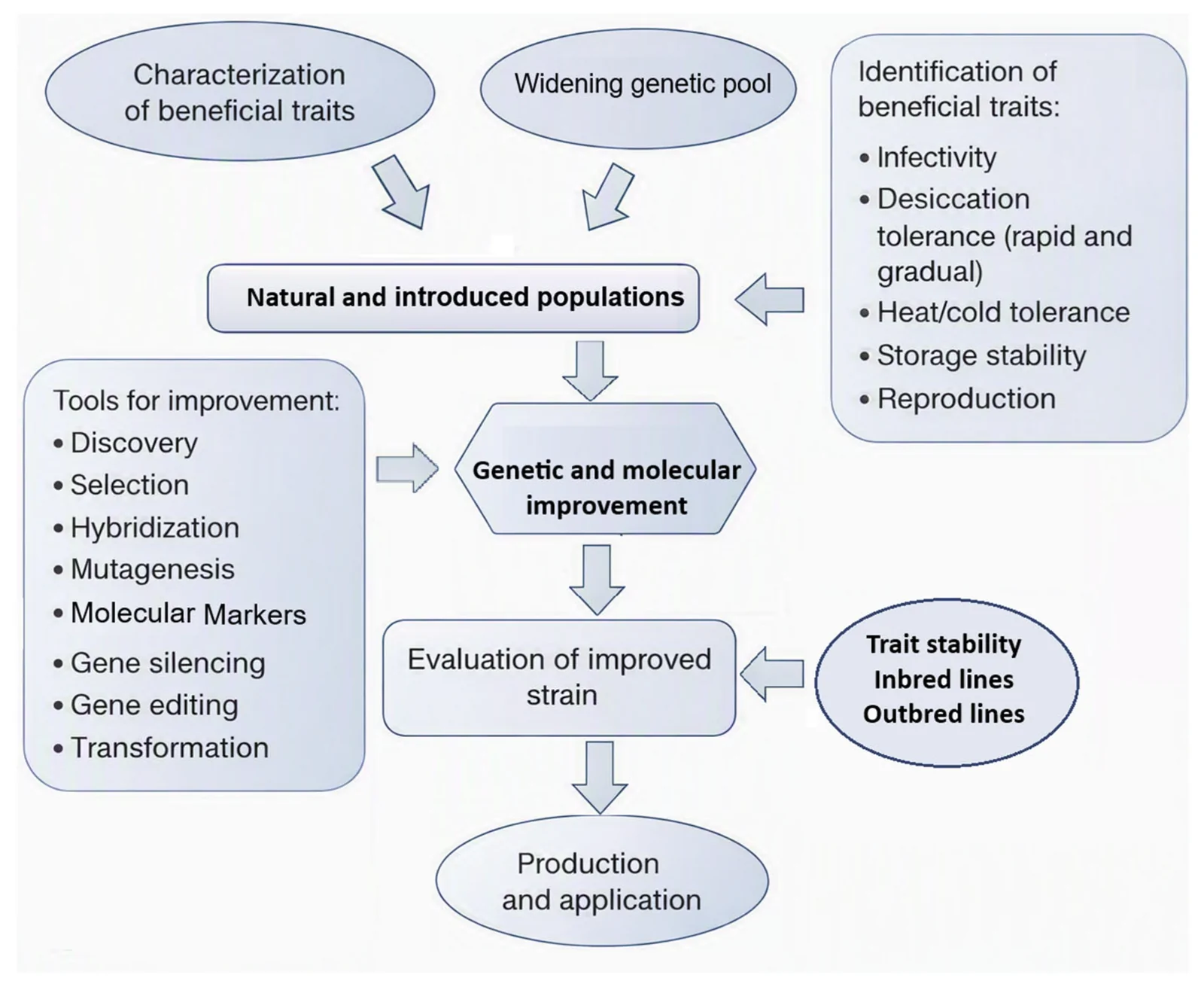
Phases and constituents for genetic improvement of entomopathogenic nematodes (redrawn from Glazer and Shapiro-Ilan [12]).

**Table 1:** Examples of molecular methods used with their purpose and contribution to entomopathogenic nematode (EPN) improvement, as well as the main limitation and validation level of their applications.

Method	Examples	Purpose and Contribution	Limitation and Validation Level	Ref.
CRISPR-Cas9	Aids in enabling high-throughput gene editing at the desired scale for *Steinernema hermaphroditum*	Use a custom guide molecule to find a specific DNA target and a protein enzyme to cut it	Complex biology and reproductive systems of EPNs; contribute at functional and phenotypic validation levels	[53]
RNA interference (RNAi)	Conducted gene *flp-21* knockdown in *S*. *carpocapsae* IJs	To study gene function or silence targeted genes	Inefficient or highly variable dsRNA delivery; in vivo laboratory validation level	[109]
RNA sequencing	Identify factors involved in EPN symbiosis with *Photorhabdus* bacteria	Mapping and quantifying the entire EPN-active transcriptomes, identify trait-specific genes, and decode complex symbiosis	Lack of spatial and single-cell resolution; high gene expression captured by RNA-seq may be affected by rapid RNA dynamics; sets transcriptome discovery and profiling validation level	[135]
Whole-genome sequencing	Insights into genetic variation and biogeography of EPNs	Secured precise taxonomy and phylogenetic set even at EPN strain level	Difficult to resolve complex structural variants and highly repetitive genomic regions; EPN strain resolution level	[52]
Starch gel electrophoresis	Revealed isozymes in *S. glaseri* that were qualitatively and quantitatively different from those in *S. carpocapsae;* distinguished between 22 strains of different *Heterorhabditis* spp.	Separates certain native proteins and isozymes like esterase and alkaline phosphatase based on a combination of charge and size	Lower overall resolution and discriminatory power than other relevant methods; introductory (internal) validation level	[136,137]
Polyacrylamide gel electrophoresis (PAGE)	Discriminated between five isolates (=four *Steinernema* spp.); morphologically, *S. glaseri* and *S. anomali* showed dissimilar/distinctive enzyme patterns revealed by PAGE	Offers superior, sharp resolution and reproducibility; it separates small-to-medium DNA/RNA fragments and proteins across a vast molecular weight range	Less rapid and has inferior resolution relative to cellulose acetate electrophoresis; introductory validation level	[138,139]
Cellulose acetate electrophoresis	Proved invaluable for identifying isolates of steinernematids, provided that a combination of enzymes is utilized	A simpler, more rapid type of electrophoresis, regarded by some authorities as being more accurate and providing superior resolution to starch gel or PAGE	Lower resolution relative to modern gels, high vulnerability to drying out and heat production due to low buffer holding capacity, high electrical current risks causing protein denaturation, and unsuitability for preparative recovery of samples; internal validation level	[35,140]
Two-dimensional electrophoresis (2-DE)	Detected more than 400 protein spots of *S. feltiae*; ten showed changes in abundance upon desiccation, and one identified as the chaperonin heat shock protein 60	Separates complex EPN protein extracts into distinct spots using isoelectric focusing and size; detects post-translational modifications in EPN proteins at relatively low cost; allows direct visual and software-based comparison of protein expression shifts between active and inactive EPN states	Matching spots across different EPN sample runs is difficult due to gel-to-gel variations; requires careful manual handling and optimization for consistent spot detection; internal validation level	[141]
Restriction fragment length polymorphism (RFLP)	Accurate and reproducible in characterizing *Steinernema* and *Heterorhabditis* species; can reveal the presence of different strains within EPN species to indicate genetic variability due to geographical origin or insect host	High reproducibility with no prior sequence needed; reliable and robust at a relatively low cost	High DNA quantity/quality needed; modern direct sequencing of ITS regions or mitochondrial DNA is faster, more accurate, and provides finer resolution than analyzing gel electrophoresis banding patterns; internal validation level	[38]
Random amplified polymorphic DNA (RAPD)	Discriminate among and within *Steinernema* spp. and *Heterorhabditis* spp.	Enables measurement of genetic diversity in EPNs, even if no prior sequence information is available; cost-effective and fast with low DNA Input	Bands show dominant inheritance (cannot tell heterozygotes apart from homozygotes); not locus-specific with low reproducibility; internal validation level	[142]
Denaturing gradient gel electrophoresis (PCR-DGGE)	Evaluate nematode biodiversity, separate species of *Steinernema*, and profile associated bacterial communities using 18S and 16S rDNA markers	Allows for simultaneous comparison of multiple EPN or soil sample profiles directly on a single gel; individual fingerprint bands can be excised, re-amplified, and sequenced for exact taxonomic identification (high throughput, cheaper, and faster for preliminary biodiversity screening)	The system clearly separated PCR fragments from 6 species except *S. tami* and *S. carpocapsae*, which had similar melting behavior despite being widely separated on the phylogenetic tree (low detection sensitivity with limited fragment size of short DNA segments); internal validation level	[143]
Amplified fragment length polymorphism (AFLP)	Distinguish genetics, persistence, and strain differentiation of 11 *S*. *feltiae* populations for their genetics, persistence, and strain differentiation	No prior sequence data required; high multiplex ratio (generates a large number of informative loci) with high reproducibility and sensitivity	AFLP markers are typically too dominant to easily differentiate between homozygous and heterozygous genotypes at a specific locus; require high-quality DNA, require expertise, and are costly; internal validation level	[43]
Microarrays	Revealed 1185 differentially expressed genes (DEGs) containing 469 down- and 716 upregulated genes in trait deteriorated *H*. *bacteriophora*; analysis of the DEGs showed that trait deterioration involves massive changes of the transcripts encoding enzymes involved in metabolism, signal transduction, virulence and longevity	Diverse genetic features are examined in one test; high-throughput capacity and high specificity for identification; identifies genes responsible for trait deterioration or desiccation tolerance during EPN commercial mass production	Detect known sequences present on the chip, missing novel genes or unexpected variations; modern high-throughput RNA-seq provides broader dynamic range and deeper discovery without requiring prior genomic sequence layouts; internal validation level	[144]
Simple sequence repeats (SSRs)	Twenty SSR loci were identified from *H*. *bacteriophora*-genomic DNA enrichment and expressed sequence tags; the values of observed and expected heterozygosities ranged from 0 to 0.536 and from 0.223 to 0.616, respectively	Detect extensive length variations and multiple alleles per locus, offering high discriminatory power to differentiate closely related EPN strains or geographical isolates with high reproducibility and low DNA quantity; distinguish between homozygous and heterozygous conditions, making it easier to study the genetic makeup and mating systems of nematode populations	Expensive and labor-intensive for uncommon EPN species; misinterpretation of heterozygosity if non-amplification occurred; homoplasy may happen via convergent mutation; can serve internal or external validation level	[145]
Inter-simple sequence repeats (ISSRs)	Distinguished 3 different isolates that belong to the same *Heterorhabditis* species from different fields in Egypt	Simple protocol with no prior sequence needed; produce multiple informative loci per reaction (so distinguishing closely related EPN strains or isolates) and not costly	Cannot easily distinguish heterozygotes from homozygotes (dominant markers); sensitive to PCR conditions, as banding profiles can vary slightly if annealing temperatures or DNA template concentrations are not strictly optimized; can serve internal or external validation level	[146]
Real-time PCR (qPCR)	Detected a polyspecific nematode community including EPN species and their natural enemies for better grasping of soil food web	High sensitivity that detects very low DNA quantities with high speed and throughput while reducing cross-contamination risks	Detects only predefined species based on chosen primers, even extracellular DNA from dead EPNs is detected; costly; external validation level	[116]
Multiplex PCR	Used more sets of primers/primers in the same qPCR reaction, as various species need to be screened in the same sample to unveil their sympatric distributions	Detects more than one species simultaneously; quick and inexpensive	Need species-specific primers and optimization of reagent concentrations and the given settings; internal or intermediate validation level	[120]
Internal transcribed sequences (ITSs)	Related sequences are useful markers for EPN identification, setting *Heterorhabditis* spp. into three groups, namely Indica, Bacteriophora, and Megidis	Rapid and accurate with high reproducibility; pairwise analyses allowed the assessment of intraspecific and interspecific sequence variability within most of the species and strains	As ITS evolves rapidly under low evolutionary pressure, it works poorly for resolving deeper evolutionary relationships among divergent EPN lineages; can serve internal or external validation level	[147]
DNA sequencing	DNA sequences were analyzed using the BioEdit ver 7.2.6 program and BLAST programs; homology and phylogeny analyses were carried out to determine the genetic relationship of nematode isolates with isolates from other countries found in GenBank; four species of EPNs were obtained from different rhizospheres	Distinguish EPN species that are morphologically similar, using different EPN developmental stages. Ease in detecting target genes related to virulence, heat tolerance, and environmental persistence for selective breeding; can set evolutionary relationships and clarify genetic diversity within strains	Depend on reference sequence databases (e.g., GenBank); mislabeled public entries can lead to misidentifications; costly and requires expertise, does not measure phenotypic fitness of EPN; can serve internal or external validation level	[148]

## 5. Conclusions

Growers and stakeholders will expand the use of EPNs and their symbiotic bacteria for pest control only when their application is more reliable and profitable than alternative tactics. One of the most meaningful transitions leading to such an adoption of EPN products is to improve EPN-biocontrol traits against key crop pests. The gained molecular knowledge, technologies, and materials developed for EPNs and their symbiotic bacterial research can be harnessed for their improvement. Remarkable examples have clearly shown that the use of traditional and emerging genetic techniques can significantly enhance EPN performance. Such techniques involve discovery, selection, hybridization, and transgenics. Transcriptomic investigation enabled the characterization of genes and molecular markers as genetic selection devices for raising EPN performance, even under environmental stresses. Predictions of ligand–target interactions for the secondary metabolites of the symbionts against specific proteins of the host pests should be expanded for biocontrol plans that save time, money, and effort. Moreover, modern molecular tools allow rapid and fine-scale taxonomic resolution for identification and quantification of EPNs. Yet, the pros and cons of molecular techniques should be known for their sound and optimal use. For example, RFLPs, SSRs, and SNPs have co-dominant molecular markers that allow a technician to distinguish heterogeneity from homogeneity in an EPN population, but AFLPs have dominant molecular markers that generally do not. Furthermore, a few molecular techniques possess superior utility in optimizing EPN applications. Metabarcoding has fine-scale taxonomic resolution to distinguish closely related EPN species that have divergent phenotypes for key traits like habitat adaptation and insect host specificity. Also, qPCR combined with traditional methods was used to indicate complicated soil biotic interactions and identify the abiotic factors that negatively or positively modify EPN effectiveness. Moreover, understanding the molecular processes behind EPN efficiency and fitness trade-offs is critical for optimizing biological pest control, engineering resilient biocontrol agents, and reducing the economic costs of mass production. Using such molecular tools should be expanded to advance our efforts toward achieving durable and eco-friendly agricultural production.

## Figures and Tables

**Figure 1:**
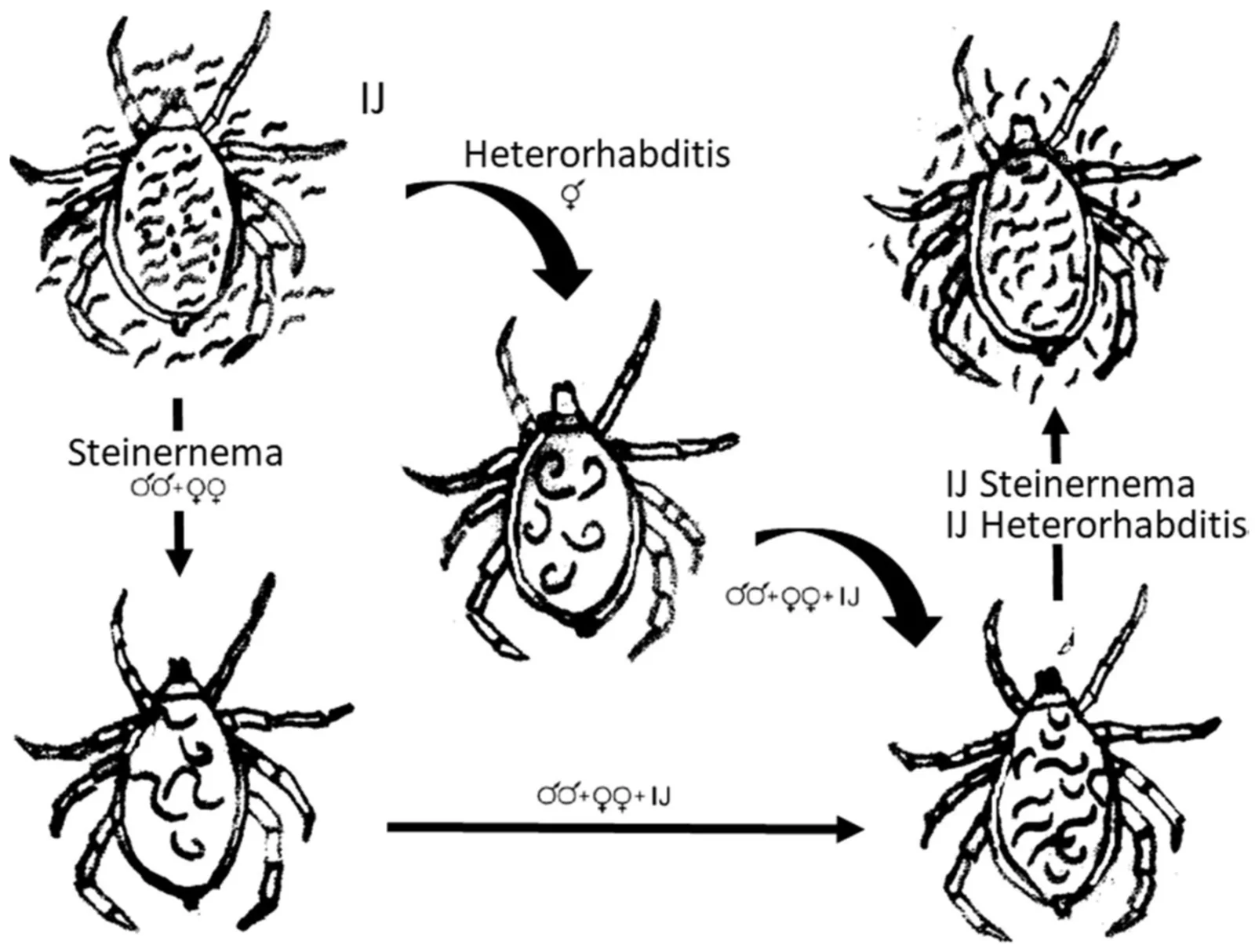
Schematic nematode life cycle showing differences in biological development between species of *Steinernema* and *Heterorhabditis* reproducing on their hosts; redrawn from Tarasco et al. [10].

## Data Availability

No new data were created or analyzed in this study. Data sharing is not applicable to this article.
